# The neural correlates of inhibitory control in 10-month-old infants: A functional near-infrared spectroscopy study

**DOI:** 10.1016/j.neuroimage.2022.119241

**Published:** 2022-05-07

**Authors:** Abigail Fiske, Carina de Klerk, Katie Y.K. Lui, Liam Collins-Jones, Alexandra Hendry, Isobel Greenhalgh, Anna Hall, Gaia Scerif, Henrik Dvergsdal, Karla Holmboe

**Affiliations:** a Department of Experimental Psychology, University of Oxford, Oxford, United Kingdom; b Department of Psychology, University of Essex, Essex, United Kingdom; c Department of Psychology, University of Bath, Bath, United Kingdom; d Department of Medical Physics & Biomedical Engineering, University College London, London, United Kingdom; e Department of Psychology, University of Cambridge, Cambridge, United Kingdom; f Institute of Mental Health, University College London, London, United Kingdom; g Nord University Business School, Department of Entrepreneurship, Innovation and Organisation, Bodø, Norway; h School of Psychological Science, University of Bristol, Bristol, United Kingdom

**Keywords:** Executive function, Response inhibition, Infancy, Functional near-infrared spectroscopy, Prefrontal cortex, Parietal cortex

## Abstract

Inhibitory control, a core executive function, emerges in infancy and develops rapidly across childhood. Method-ological limitations have meant that studies investigating the neural correlates underlying inhibitory control in infancy are rare. Employing functional near-infrared spectroscopy alongside a novel touchscreen task that measures response inhibition, this study aimed to uncover the neural underpinnings of inhibitory control in 10-month-old infants (*N* = 135). We found that when inhibition was required, the right prefrontal and parietal cortices were more activated than when there was no inhibitory demand. This demonstrates that inhibitory control in infants as young as 10 months of age is supported by similar brain areas as in older children and adults. With this study we have lowered the age-boundary for localising the neural substrates of response inhibition to the first year of life.

## 1 Introduction

The early years of life represent a fundamental period in the development of executive functions (EFs) - a set of core cognitive skills, such as inhibitory control, working memory, and cognitive flexibility, that develop slowly from infancy and improve rapidly in early childhood (Hendry et al., 2016). Advances in these early cognitive skills are accompanied by important maturation processes in the prefrontal cortex (PFC), a region of the brain consistently associated with EFs (for reviews, see Diamond, 2002; Fiske and Holmboe, 2019). One important form of inhibitory control is response inhibition; the ability to inhibit a prepotent or well-learned response (Friedman and Miyake, 2004). Whilst it is possible to reliably measure response inhibition from the second half of the first year of life (Diamond, 1985; Hendry et al., 2021; Holmboe et al., 2008, 2018, 2021), very little is known about the brain mechanisms supporting response inhibition in infancy. The reason for this is twofold: a lack of age-appropriate tasks (Holmboe et al., 2021) and the difficulty in using neuroimaging techniques for measuring the awake infant brain in non-clinical settings (Lloyd-Fox et al., 2010). The current study aims to identify the functional neural correlates of inhibitory control, an essential EF component, as it emerges in the first year of life.

Canonical tasks such as the Stop-Signal (Logan and Cowan, 1984) and the Go/No-Go task (e.g., Drewe, 1975) have frequently been used with older children and adults alongside functional magnetic resonance imaging (fMRI) to investigate the brain regions associated with response inhibition (Aron et al., 2007; Aron and Poldrack, 2006; Booth et al., 2003; Cope et al., 2020; Durston et al., 2002; Hampshire et al., 2010; Wager et al., 2005). These well-established experimental paradigms reliably measure the neural correlates of response inhibition but are unsuitable for the assessment of response inhibition skills as they emerge in infancy. This is because many of these tasks have high working memory and language comprehension demands (Holmboe et al., 2021) and neuroimaging methods such as fMRI are often not suitable for use with awake infants and young children.

To measure response inhibition in the first two years of life, researchers have used variations of the classic A-not-B task (Diamond, 1985; Diamond and Goldman-Rakic, 1985; Jacobsen, 1935; Piaget, 1954). In the A-not-B task, the researcher places – in view of the infant – a desirable object in one of two identical wells (‘A’), which are then covered. The infant is subsequently allowed to retrieve the object. This sequence is repeated to build up the infant’s prepotent reaching response to ‘A’. After several repeat hidings at ‘A’, the object is hidden in the alternate location (‘B’), such that a new response (reach to ‘B’) must be initiated, and the competing prepotent response (reach to ‘A’) inhibited. A variable delay between the hiding event and the infant’s search is usually imposed, and therefore the canonical version of the A-not-B task has both a working memory and a response inhibition component (Diamond, 1985, 2002; Holmboe et al., 2018). In a seminal study by Diamond (1985), it was established that 6–12-month-olds perseverate on this task with varying delays, i.e., they continue to search at ‘A’ even when they have just seen the toy hidden at ‘B’.

The imposed delay in the canonical version of the A-not-B task is problematic if the main aim is to measure response inhibition alone (i.e., without a working memory component). However, even versions *without* a hiding event (e.g., the location is simply cued by waving the cover for the well; Smith et al., 1999; Clearfield et al., 2006) or with no delay (infant is allowed to reach immediately; Hendry et al., 2021) produce a robust perseverative response in 8–16-month-old infants. This suggests that it is possible to build up a prepotent response in infants, and to assess response inhibition by measuring the ability to overcome that prepotent response, without taxing working memory. The task used in the current study, the Early Childhood Inhibitory Touchscreen Task (ECITT; Holmboe et al., 2021), was designed on this principle of building up a prepotent response that then must be inhibited, but rather than hiding or cueing objects, it uses an engaging touchscreen interface. As with the A-not-B task, the ECITT has been found to elicit perseverative responses, presumed to be attributable to a failure of response inhibition, in children aged 10 months and upwards (Hendry et al., 2021; Holmboe et al., 2021).

A number of studies have used a ‘looking version’ of the A-not-B task (where the trigger for the reward is the infant visually fixating on the correct location rather than reaching to it) alongside electroencephalography (EEG) to investigate inhibition-related brain activation in infancy (Bell and Fox, 1992; Bell and Wolfe, 2007; Cuevas et al., 2012; although note that Bell and Fox, 1992 used a reaching version of the task). These studies have identified broad indices of neural activation during response inhibition, such as alpha power over frontal channels or changes in EEG power from baseline in frontal regions. Whilst these studies have provided important insights into the brain mechanisms underpinning inhibition, the limited spatial resolution of EEG has meant that further localisation within frontal regions (and other regions of the cortex) has not been possible. Also, many of these EEG studies have employed passive, visual-only response inhibition tasks that require no motoric response from the infant – in contrast to the fMRI literature with older children and adults, where canonical response inhibition tasks requiring a motoric response are used as a behavioural index. As such, we presently have limited knowledge of which specific areas of the brain are activated when infants engage in active inhibitory control involving a motor response.

The relatively recent addition of functional near-infrared spectroscopy (fNIRS) into the developmental researcher’s toolkit has made it possible to investigate neural activation in specific brain areas during the early stages of development (Fiske and Holmboe, 2019; Lloyd-Fox et al., 2010; Wilcox and Biondi, 2015). fNIRS is a non-invasive, optical imaging technique that uses near-infrared light to measure changes in the concentration of oxygenated and deoxygenated haemoglobin in the blood in the cortex. An early fNIRS study by Baird et al. (2002) illustrates the role that fNIRS can play in better understanding the neural correlates of cognitive processes in infancy. This study demonstrated the involvement of the prefrontal cortex in short-term memory in 5−12-month-old infants. More recently, multiple studies have used fNIRS to investigate the neural correlates of EF in preschool and school-age children (Buss et al., 2014; Kerr-German and Buss, 2020; Monden et al., 2015; Moriguchi et al., 2018; Yanaoka et al., 2020; Zhou et al., 2022), evidencing the feasibility and growing popularity of this technique in developmental research. Whilst studies have begun to provide insight into the neural mechanisms involved in the development of working memory in infants and toddlers (Delgado-Reyes et al., 2020; Wijeakumar et al., 2019), few studies have identified the specific areas of the brain that are functionally associated with inhibitory control in children under 2 years of age.

In research with children and adults, studies using fNIRS have found activation in the right PFC and parietal cortex during task trials where inhibition was required; although children (4 – 6 years) showed a more immature response pattern (Mehnert et al., 2013). Similarly, research has found increased right lateral PFC activation in pre-schoolers ($3\frac{1}{2}-4\frac{1}{2}$ years) when response inhibition was successful (Moriguchi and Shinohara, 2019). This is consistent with fMRI and transcranial magnetic stimulation research showing that regions of the PFC and parietal cortex are activated during tasks of inhibitory control in both adults and children (Bunge et al., 2002; Cope et al., 2020; Durston et al., 2002, 2006; Tamm et al., 2002; van Belle et al., 2014). Specifically, the right dorsolateral prefrontal cortex (DLPFC; Casey et al., 1997; Cope et al., 2020; Durston et al., 2006), the right inferior frontal cortex (as part of a frontobasal-ganglia network; Aron et al., 2004, 2014; Bari and Robbins, 2013; Chambers et al., 2009; Chikazoe, 2010), and regions within the right parietal cortex (Bunge et al., 2002; Chikazoe et al., 2009; Cope et al., 2020; Durston et al., 2002; Gonzalez Alam et al., 2018; Mehnert et al., 2013; Osada et al., 2019) are reliably found to be active in tasks requiring response inhibition. Some studies have also found activation in the bilateral orbito-frontal cortex when response inhibition is needed (Casey et al., 1997; Cope et al., 2020; Rubia et al., 2006; Tamm et al., 2002). With this current study, we aimed to investigate whether similar patterns of brain activation can be observed in infancy during a response inhibition task.

### 1.1 The current study

The overarching aim of this study is to elucidate the neural under-pinnings of response inhibition in 10-month-old infants by using fNIRS alongside a recently developed task suitable for this young population. We used a version of the Early Childhood Inhibitory Touchscreen Task (ECITT; Holmboe et al., 2021), in which infants are required to inhibit their response to a prepotent (frequently rewarded) location and instead make a response to an alternative location to receive the reward (a short animation). The ECITT overcomes many methodological barriers that are problematic in other tasks by ensuring minimal language and working memory requirements, and including fun animations that maximise task engagement in this age group (Holmboe et al., 2021). Importantly, the task has been shown to elicit inhibitory effects behaviourally in infants as young as 10 months of age (Hendry et al., 2021). Since a manual motoric response is required from infants, this task more closely mirrors the structure and demands of canonical response inhibition tasks used with older participants in the fMRI/fNIRS literature.

In this study we adopted a mixed exploratory and confirmatory approach. Based on findings in older children and adults, we designed our fNIRS probe to cover the bilateral PFC and the bilateral area around the intraparietal sulcus. However, due to the lack of existing fNIRS studies that use manual response-based tasks and the very limited prior evidence of neural activation during response inhibition in infant populations, we did not pre-specify hypotheses about which specific brain regions would be associated with response inhibition at this age. Instead, in our group-level analyses, we first identified channels demon-strating a significant haemodynamic response and, within these, looked for channels in which the haemodynamic response was differentiated by block type (e.g., inhibitory-demanding ‘experimental’ blocks vs. ‘control’ blocks with no inhibitory requirement). We also identified the time course of the significant effects. After this, in a set of pre-registered analyses (https://osf.io/qs4h8) using the pre-identified channels and time windows, we investigated whether neural activation was associated with individual differences in response inhibition performance in 10-month-old infants.

## 2 Materials and methods

### 2.1 Participants

Participants were 138 10-month-old infants (67 males) recruited for a longitudinal study on early EF development. Three male infants were excluded due to birth complications leading to health-related concerns. Data presented in this study were collected in the second of two visits to the Oxford University Babylab, spaced approximately one week apart. In the first session, infants completed a behavioural version of the ECITT amongst other tasks. Parents gave informed consent before participating with their child and received a £20 online shopping voucher and small Babylab branded gift for their participation. The longitudinal study received ethical approval from the University of Oxford Central University Research Ethics Committee: R57972/RE010. See Supplementary Materials 1 (SM 1) for further details of the longitudinal study, including sample demographics (infant age, ethnicity, and socioeconomic status indicators) and study inclusion criteria. Following a comprehensive data processing pipeline (see Section 2.6), the final sample of participants with valid behavioural data was 121 infants, of whom 59 also contributed valid fNIRS data. Full details of data exclusions are provided in Section 2.5.3.

### 2.2 Apparatus

**Fig. 1:**
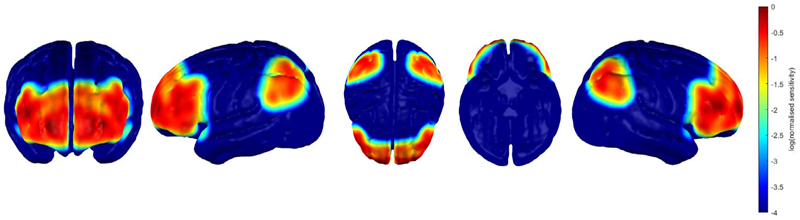
fNIRS Array Sensitivity Map *Note*. This sensitivity heat map was generated on a 12-month-old infant head model (Shi et al., 2011) using the optode positions associated with this probe. These maps are scaled to the maximum sensitivity value in the grey matter mesh and are displayed on a log normalised scale. From left to right: frontal view, left view, superior view, inferior view, and right view of the sensitivity of channels in this array.

**Fig. 2:**
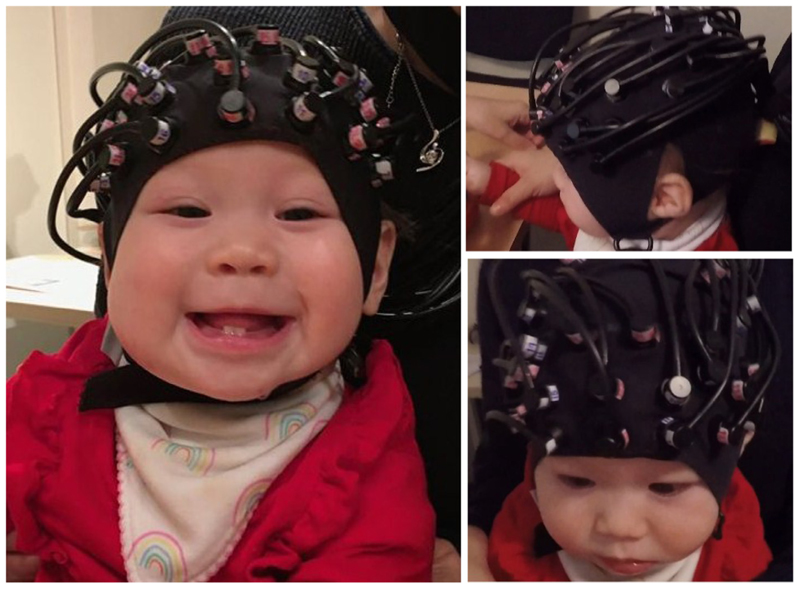
fNIRS Cap Placement *Note*. Consent for the publication of this image has been obtained from the participant’s parent.

fNIRS data were collected with the Gowerlabs NTS continuous wave system at a sampling rate of 10 Hz. Near-infrared light was emitted at 780 and 850 nm to measure changes in concentration of deoxygenated (HHb) and oxygenated (HbO_2_) haemoglobin, respectively. The source levels were manually set, most commonly to 80% intensity (60% for short separation channels), however this varied depending on the infant’s amount and type of hair. The probe geometry consisted of 32 optical sensors with a source-detector distance of 25 mm (including two 12 mm ‘short’ channels) that were fitted to an EasyCap. The probe was anchored onto three 10–5 reference points: FpZ, P1 and P2. A total of 46 channels covered the bilateral PFC and area around the intraparietal sulcus, see Fig. 1 for a sensitivity map that indicates the approximate neural areas covered by our array. The headgear was fitted to the infant’s head so that the front of the cap sat just above the eyebrows, and the optode anchored to FpZ was positioned centrally between the eyebrows, see an example in Fig. 2. See also SM 2a for further cap placement information and SM 2b for a channel map.

### 2.3 Early Childhood Inhibitory Touchscreen Task (ECITT)

**Fig. 3:**
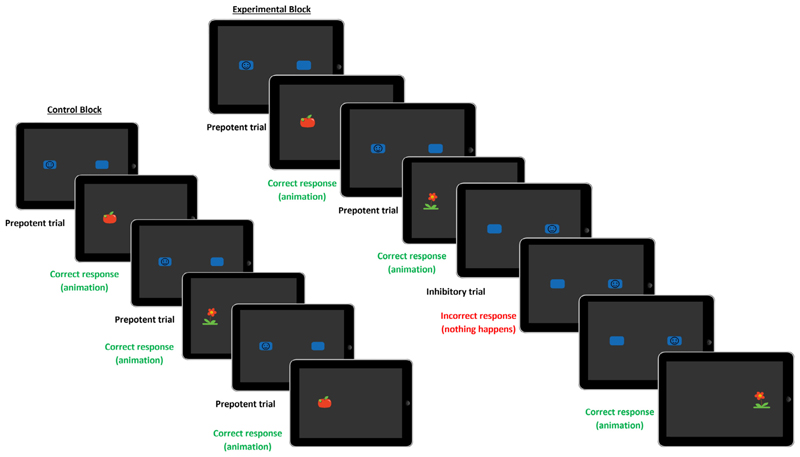
The Early Childhood Inhibitory Touchscreen Task *Note*. Left: An example sequence of trials in a control block, where all trials were prepotent. Right: An example trial sequence in an experimental block, where 50% of trials were inhibitory.

In the ECITT, infants are presented with two blue ‘buttons’ on the left and the right of a touchscreen (iPad): one blank, and one with a smiley-face icon (see Fig. 3). Tapping the button with the smiley-face icon triggered a 4-second animated cartoon. The smiley-face icon occurred more frequently in one location (prepotent trials; 75%) than the other (inhibitory trials; 25%). Therefore, on inhibitory trials, infants must inhibit their response to the prepotent location and instead make a response to the other location to trigger the animation. In the first test session, infants completed the ‘behavioural’ version of the task, as described by Hendry et al. (2021). Further details about the method, as well as the results from the behavioural version of the task administered in the first test session, are reported in SM 5a. Analyses of consistency between performance on the behavioural and fNIRS versions of ECITT are reported in SM 5b.

In the second test session, participants completed a blocked version of the ECITT that was adapted for use with fNIRS, see Fig. 3 for example trial sequences (a video of an infant completing the task is available at https://osf.io/m58qs/). The fNIRS version of the ECITT was divided into control and experimental blocks (each consisting of six trials) that were separated by a neutral baseline block (moving abstract shapes accompanied by calm music). The blocks were always presented in the same order: baseline, control, baseline, experimental, baseline, control etc. The purpose of the baseline blocks was to have a period of minimal cognitive effort (a ‘resting state’) so that we could compare the haemodynamic response during active task blocks (control and experimental blocks) with that obtained during a non-task ‘rest’ period (Wilcox and Biondi, 2015). Baseline block durations were jittered (12–17 s) to minimise anticipatory neural activation ahead of active task blocks. During control blocks the target always appeared in the prepotent location (six prepotent trials). In experimental blocks, the target appeared on the prepotent side 50% of the time (3 trials), randomised with the constraints that the target was always presented in the prepotent location on the first trial and the target could not consecutively appear in the same location more than twice. Therefore, across the blocked version, the proportion of prepotent (75%) and inhibitory (25%) trials was the same as in the behavioural version. It is expected that prepotency in the control blocks would carry over into experimental blocks, requiring response inhibition to be used on trials where the target was presented in the inhibitory location.

Since the task is response-contingent, the duration of each trial varied depending on how long the infant took to respond. No minimum or maximum trial length was imposed, although during behavioural data processing, trial lengths of ⟨300 ms or ⟩ 5000 ms were excluded from analysis. If the infant made an incorrect response, there was no change on the screen and the trial continued until a correct response was made. Once a correct response was made, the animation played, and the next trial began. This was the case in both control and experimental blocks, such that each infant received six animated cartoons in the control block and six in the experimental block. The blocked ECITT had no fixed stopping point, but infants were encouraged to complete at least three blocks of each type (experimental and control) to ensure enough reliable fNIRS data had been collected, and for as long as the infant was engaged with the task. The task was stopped if the infant became excessively distracted or distressed, or at the request of the parent. For those with valid fNIRS data (*N* = 59), the number of control blocks (all prepotent trials) completed ranged from 3 to 7 (*M* = 4.85), with a mean duration of 43 s (SD = 17 s). The number of experimental blocks (mixed prepotent and inhibitory trials) completed ranged from 3 to 7 (*M* = 4.58), with a mean duration of 49 s (SD = 18 s). The total number of blocks completed ranged from 6 to 14 (*M* = 9.43), which is equivalent to 36–84 individual trials. See Section 2.6 for details of validity criteria and data exclusions.

A note on terminology: Prepotent and inhibitory trials originate from the behavioural version of the task where we describe each trial (and there are no blocks). The control and experimental blocks contain these two trial types – the control blocks only contain prepotent trials (no inhibitory demand), whereas the experimental blocks also contain inhibitory trials (50%) and therefore do have an inhibitory demand.

### 2.4 Procedure

Before the first test session, infants were allocated a prepotent side (left or right) that was counterbalanced between infants and remained the same for all test sessions. To ensure that a prepotency was established as intended, if infants made an incorrect response on the first (prepotent) trial of the ECITT in Session 1, the experimenter re-started the task with the target appearing in the location first reached to (*N* = 21). The revised location was used as the allocated prepotent side for all subsequent test sessions (including the fNIRS session).

Infants were seated on their caregiver’s lap and positioned at a small table, adjacent to the experimenter. The iPad (presenting the ECITT) was held horizontally directly in front of the child within their reach. There were no demonstration or practice trials, however on the first trial (not included in analyses), the experimenter cued the target by pointing and instructed the child to “touch the happy face”. This was done to ensure that the prepotency was established from the start of the task. After the first trial, if the target location was cued by either the experimenter or the parent, the trial was coded as invalid (see Section 2.5.3. for details of validity criteria and data exclusions). Verbal encouragement was given when necessary to increase engagement but was kept to a minimum. During the animations and baseline blocks, the experimenter removed the iPad from the child’s reach (still within their sight) to prevent them from touching the screen and making an invalid (accidental or premature) response.

### 2.5 Data processing

#### 2.5.1 Behavioural data processing

The ECITT software automatically recorded the accuracy of responses at the trial level. However, because infants’ responses were not always detected, the accuracy and validity of each trial was reviewed manually prior to analysis (see SM 3a for details of the coding scheme). The ECITT software also automatically records response time data (ms) for each trial, which refers to the time from the start of the trial to when a response is made. For several reasons we determined that it was not appropriate to use reaction time data from infants this young in our analyses, and so instead focus our analyses on accuracy data. For further discussion of why we do not consider reaction time data from 10-month-olds valid, see Lui et al. (2021) and Hendry et al. (2021). See also SM 6e for further information and for descriptive statistics of raw (non-coded) response time data.

Videos of the testing sessions were coded offline by two coders (AF and KL) who gained excellent inter-coded reliability: *κ* = 0.92 for accuracy, *κ* = 0.85 for validity across 21 videos (833 trials). Using the Observer XT video coding software, infants’ looking behaviour during baseline blocks (described in Section 2.3) was coded offline by two trained coders (AF and KL). According to the baseline coding scheme (described in full in SM 3b), infants’ looking behaviour was coded as either: looking at the iPad screen displaying the baseline video or somewhere else neutral (e.g., the table), looking at faces (experimenter or parent) or as ‘other’ (e.g., infant was crying, fussing, or moving). Baseline blocks were deemed as valid and included in the fNIRS analysis as an indicator of the infants’ ‘resting’ haemoglobin levels (which task block activation could be compared to) if the participant looked at the baseline video or other neutral location for at least 60% of the baseline duration. Baseline blocks were coded as invalid and excluded from the fNIRS analysis during pre-processing (see Section 2.5.2) if the participant was crying, fussing, fidgeting, or looking at the face of an experimenter or parent for more than 40% of the baseline duration. Excellent inter-coder reliability was established across 41 videos (1715 coding incidences): *κ* = 0.85.

#### 2.5.2 fNIRS data processing

A description of our fNIRS setup, including information about the steps taken to convert raw data (recorded by the NTS system) into .nirs data suitable for pre-processing with event markers (start of each block, imported from the ECITT software) and timing information, can be found in SM 2c. Also see SM 2c for access to the custom MATLAB scripts used in this process.

Data were then entered into HomER2, a MATLAB based toolkit (Huppert et al., 2009), for pre-processing. Invalid blocks, baselines and non-task related data were manually excluded (see SM 4a for details of block exclusion). Raw intensity data were converted to optical density and channels with very high or very low optical density (1e-03 - 1e+03) were excluded at the participant level. Participants with more than 2/3 of channels excluded were not included in further analyses (see Section 2.5.3). Motion artifacts were identified and corrected using a spline interpolation followed by a wavelet transformation (Di Lorenzo et al., 2019). The motion-corrected optical density data were then converted to concentration changes in HbO_2_ and HHb using the modified Beer-Lambert Law with path length factors of 5.2 (HHb) and 4.8 (HbO_2_), see Scholkmann and Wolf (2013). A band-pass filter (high pass; 0.01, low pass; 0.80) was applied to remove low frequency noise and high frequency physiological signal from the data. See SM 4b for full details of parameters used in the pre-processing stream.

HbO_2_ and HHb concentration change data from each channel were block averaged over a period of 47 s, of which two seconds contained data from valid baseline blocks. Baseline concentration change data was subtracted from the average haemoglobin concentrations in the 45 s experimental window. The baseline-corrected haemoglobin concentration data were divided into nine five-second time-bins. Following investigations into the average block duration (control; *M* = 43 s, *SD* = 17 s; experimental; *M* = 49 s, *SD* = 18 s; see also SM 4a) we focused our analysis on data from 0 to 35 s of the time course. This is because, due to the variable block duration (contingent on the duration of infants’ responses), the last two time-bins captured baseline data from some participants and task data from others, see SM 7b for more information on this decision. Results with these time-bins included are reported in SM 7e and are in convergence with those reported here (Section 3.2).

Data from 42 channels (of 46) were included in statistical analyses at the group-level; two channels (Channel 11, right parietal cortex, and Channel 15, left PFC) were excluded as less than 70% of participants contributed data to these channels, and data from the two short separation channels were also excluded from analyses. The short separation channels were included in our array with the intention to filter out superficial haemodynamic response (i.e., physiological noise) measured from the scalp and skull, however these were excluded from analyses because, being positioned at a separation of 12 mm, the short channels were not optimised for short separation regression techniques (see Brigadoi and Cooper, 2015 and Emberson et al., 2016 for discussion, and see also SM 2d for the rationale behind our decision to exclude the short channels from our analysis). We instead used bandpass filtering to remove some level of systemic noise from our data (described above).

#### 2.5.3 Data exclusions

Following exclusions for completing less than 2 blocks in each condition (*N* = 13) or poor accuracy (< 60%) on the prepotent trials (*N* = 1), ECITT behavioural data was available for 121 infants; see SM 6a for prepotent and inhibitory accuracy by block type (control and experimental). After exclusions for: task refusal (*N* = 2), refusing the fNIRS cap (*N* = 2), data file loss (*N* = 1), completing less than 3 blocks in each condition (*N* = 36), contributing valid fNIRS data for less than 3 blocks in each condition (*N* = 13), and over- or under-saturation of more than 2/3 of channels (*N* = 8), ECITT fNIRS data was available for 59 infants. See SM 4a for further details on fNIRS block exclusions. The final sample of participants with valid fNIRS data was 59; thus, there was considerable data attrition (~ 51% of data excluded).

#### 2.5.4 Behavioural measures

The manually coded trial level accuracy data from the ECITT was used to generate three dependant variables: ‘prepotent accuracy’, ‘inhibitory accuracy’ and ‘adjusted AccD’. The ‘adjusted accuracy difference’ (adjusted AccD) variable was created as a measure of response inhibition using the following formula: (prepotent accuracy – inhibitory accuracy)/prepotent accuracy. The larger the difference between the accuracy score on prepotent and inhibitory trials, the larger the adjusted AccD score. A smaller difference resulted in a smaller adjusted AccD score, which is indicative of better response inhibition. The adjusted AccD is a variation of the ‘AccD measure’ (prepotent accuracy – inhibitory accuracy) that was used by Holmboe et al. (2021) in their original study. We generated the adjusted AccD score to control for instances where infants performed relatively poorly on both trial types (i.e., the adjusted score took the infants’ level of performance on the prepotent trials into account). This is important because performance on prepotent trials varies more in 10-month-olds than in the older infants and toddlers assessed in Holmboe et al. (2021). In their paper, Holmboe et al. (2021) compared the AccD and the adjusted AccD and found highly consistent results from both measures.

It is worth noting that a small number of participants (*N* = 14) obtained a negative adjusted AccD score; the majority of which had very high accuracy on both prepotent and inhibitory trials. A slight negative score is appropriate in cases where high performing infants with very little difference between prepotent and inhibitory trials may have made a few errors on prepotent trials (of which there are many more), possibly after two inhibitory trials in an experimental block where they may have begun to build a slight prepotency to the inhibitory side. The adjusted AccD measure corrects to some extent for performance on the prepotent trials, but at very low prepotent accuracy, the assessment of response inhibition ability will be less accurate due to measurement error.

In cases where there is a larger disparity between accuracy on inhibitory and prepotent scores in the ‘wrong’ direction (i.e., the infant seemingly performs better on the prepotent trials than on the inhibitory trials), we must consider the influence of other factors. Hendry et al. (2021) found evidence for a right side prepotent bias in 10- and 16-month-old infants during the behavioural version of the ECITT. That is, infants assigned to the ‘left’ prepotent side and who had a right-side bias would find it more difficult to build up a prepotency to the left. As part of our administration protocol, we took steps to minimise the impact of such side biases by switching the prepotent side if the infant made an incorrect response on the first trial, because piloting indicated that such first responses were often indicative of a side bias. Nevertheless, more subtle continuing biases cannot be ruled out (e.g., where an infant initially responds correctly to the pre-assigned prepotent location on the left, but subsequently appears to show a side bias to the right). Whilst it is important to note that a large negative adjusted AccD score only occurred for a very small number of participants (*N* = 6 out of 121, see SM 6b), future research should investigate the potential impact of side biases in large participant samples (as this performance pattern is rare).

### 2.6 Statistical analysis

All analyses performed as part of this study were conducted in SPSS version 27. Confidence intervals (CI; 95%) were calculated on 1000 bootstrap samples. All variables were tested against the parametric test assumptions, and when these were violated, appropriate non-parametric equivalents were also used to test for convergence. Data distribution plots, details of assumption tests, and the results of the equivalent non-parametric tests are reported in SM 5 (Session 1; ‘Behavioural’ ECITT), SM 6 (Session 2; ECITT with fNIRS) and SM 7c and 7d (pre-registered individual differences analyses). For the fNIRS data, Greenhouse-Geisser corrected degrees of freedom and significance values were used because the channel-level data did not meet the sphericity assumption required for repeated measures ANOVA. Where multiple tests were conducted throughout the study, the procedure for controlling the false discovery rate (FDR; Benjamini and Hochberg, 1995) was used.

#### 2.6.1 Behavioural analyses

Of the 121 participants with valid ECITT data, 59 also had valid fNIRS data (sub-sample A) and 62 participants only had valid behavioural data (sub-sample B). Results of an independent *t*-test revealed no significant difference in adjusted AccD scores between sub-sample A (*M* = 0.402, *SD* = 0.332) and sub-sample B (*M* = 0.371, *SD* = 0.340); *t* (119) = 0.497, *p* = .620, *d* = 0.336. This result demonstrates that there were no significant performance differences between those with, and without, valid fNIRS data.

Performance of the 112 infants who had valid ECITT data from both sessions (behavioural version of ECITT in Session 1, and the blocked ECITT with fNIRS in Session 2) were compared. Paired *t*-tests indicated that there were no significant changes in adjusted AccD scores across the two test sessions; *t* (111) = 0.073, *p* = .942, *d* = 0.007, and results of Pearson’s correlation analyses of adjusted AccD scores demonstrate that infants performed consistently across sessions; *r* (110) = 0.482, *p* <0.001, [*CI* = 0.296, 0.638]. Tests were also run with the prepotent and inhibitory accuracy variables and are reported in SM 5b. Results demonstrate consistency in performance across sessions.

#### 2.6.2 fNIRS group-level analyses

Using a custom MATLAB script (https://osf.io/mpt5g/), the block averaged haemoglobin concentration data were organised such that there was a separate variable for each channel, time-bin, chromophore, and block type (control, experimental, baseline), following the approach taken by de Klerk et al. (2018) and Lloyd-Fox et al. (2015). To identify channels demonstrating a significant haemodynamic response (increase in HbO_2_ and/or HHb decrease) from baseline over time (main effect of time), repeated measures ANOVAs (per channel) were conducted with time bin (7 levels) and condition (2 levels) as within-subject factors. In the analyses, a significant main effect of time would indicate significant haemodynamic concentration changes from baseline across the experimental window (35 s). For those channels demonstrating a significant haemodynamic response over time, repeated measures analyses were conducted to investigate whether this response differed between experimental and control blocks, as indicated by a significant main effect of block type or a significant time × block type interaction.

#### 2.6.3 Time course of the block type effect

After channels showing a significant block type effect had been identified (following the process described above), paired *t*-tests were conducted in each 5-second time-bin (0 – 35 s) on haemoglobin concentration data from each channel (in channel space) that showed a significant block type effect in either the HbO_2_ or HHb signal. The purpose of this analysis was to examine the time course of the significant block type effect (change over time-bins) and so ‘time-bin’ is treated as a categorical variable for each planned comparison.

The paired *t*-tests were run between matched variables; for example, HHb data from time-bin X of Channel Y during the control block was compared to HHb data from time-bin X of Channel Y during the experimental block. When we were examining adjacent channels as a channel pair (see Section 2.6.5), HbO_2_ or HHb data from two channels were averaged (mean) within each time-bin.

#### 2.6.4 Individual differences analyses

After we conducted our group-level analyses and time course investigation (as described above), we pre-registered our hypotheses and analysis plan for the individual differences analyses (https://osf.io/qs4h8). The purpose of these analyses was to investigate associations between the fNIRS data and infants’ response inhibition. We stated in our pre-registration that we would conduct both parametric and non-parametric tests (as we did not know the distribution of our data) and subsequently the variables were tested against the parametric assumptions. Whilst some variables were skewed (see SM 7c for details), these were skewed within an acceptable level and therefore to ensure that comparisons could be made easily between correlations, we decided to report the parametric results for all tests in the manuscript (in Section 3.3. below) and the equivalent non-parametric tests in SM 7c and 7d. Results of the parametric and non-parametric tests are consistent.

As stated in our pre-registration, we conducted three one-tailed confirmatory correlational analyses to investigate whether individual differences in neural activation in specific channels or channel-pairs across the identified time-bins were positively associated with individual performance differences in infants’ inhibitory score. We also pre-registered that we would perform exploratory correlational analyses to investigate associations between neural activation in pre-identified channels with inhibitory performance (reported in Section 3.3 and described in the pre-registration). Our justifications for running this exploratory test are outlined in the ‘Exploratory analysis’ section of the pre-registration.

Channels that were identified in our group-level analyses as showing a significantly larger increase in HbO_2_ concentration and/or a significantly larger decrease in HHb concentration in experimental compared to control blocks (results are reported in Section 3.2) were used as indices of neural activation. We calculated the difference between HbO_2_ concentrations in experimental and control blocks (experimental minus control; ‘HbO_2_ difference’), and the difference between HHb concentrations in control and experimental blocks (control minus experimental; HHb difference) and created an average of this difference measure across the identified channels or channel pairs. An ‘inhibitory score’ variable (generated using the following formula: 1 – adjusted AccD) was used as an index of infants’ response inhibition performance, such that a larger score was indicative of better response inhibition ability. The adjusted AccD score was reversed to allow us to consider positive associations with brain activation in the experimental block (compared to the control block), rather than negative associations. The reversal only changes the sign of the correlation compared to using the adjusted AccD.

#### 2.6.5 Head modelling, channel localisation & image reconstruction

To allow us to visualise the data on an age-appropriate head template, a model of the infant head was produced from averaged structural MRI data of a 12-month-old cohort (Shi et al., 2011); see SM 2e for a full description of this process. As demonstrated by Collins-Jones et al. (2021), assuming a constant head size and array position is a valid approach for an image reconstruction approach using infant fNIRS data. The head model was scaled to the group mean head circumference measurement of the 59 infants in this study with useable fNIRS data. The positions of sources and detectors were registered virtually to the scalp surface of the head model using the Homer2 spring relaxation mechanism (Aasted et al., 2015). To model the transport of near-infrared light through the head model, TOAST++ ((Schweiger and Arridge, 2014), see http://toastplusplus.org) was employed to produce a forward model for each wavelength. Using the group-level block-averaged optical density data, the forward model was then used to reconstruct a time-series of images of HbO_2_ and HHb concentration changes for each condition. Image reconstruction was constrained to the grey matter nodes of the volume mesh, as per previous topographic approaches (Boas et al., 2004; Boas and Dale, 2005). The resulting reconstructed images were mapped to the grey matter surface mesh. Data preparation, meshing, forward modelling, and reconstruction were facilitated by the DOT-HUB Tool-box (www.github.com/DOT-HUB).

The cortical positions for channels showing significant experimental effects (reported in Section 3.2) were determined using the forward model. For each channel, the sensitivity values from the forward model mapped to the grey matter surface were used to compute a weighted average of grey matter node positions; the nearest grey matter node to the weighted average position was determined. Using the infant automated anatomical labelling (AAL) atlas presented by Shi et al. (2011), the anatomical label of the nearest grey matter node was determined and was assigned as the cortical label of the channel. From this localisation process, pairs of significant channels that covered the same region of the brain were formed. This was done so that we could investigate the time course of the haemodynamic response in the channel pair associated with each region of the brain (see Section 3.2.2. for the results of the time course analysis).

Finally, *t*-statistic maps were produced comparing the group-level response to the experimental block to the response to the control block (experimental – control). Statistical mapping was conducted in the space of the grey matter surface mesh. The responses were compared between the two block types in time windows at 10 – 15 s, 15 – 20 s and 20 – 25 s of the block time course for the HbO_2_ response, and 10 – 15 s, 15 – 20 s, 20 – 25 s and 25 – 30 s for the HHb response. For each time window and for each block type, all concentration change values within the block time course across participants were concatenated to produce a single vector for each node present in the grey matter surface mesh. Experimental block and control block vectors from equivalent time windows were compared using a two-tailed paired-sample *t*-test.

## 3 Results

### 3.1 Behavioural results

In both sub-sample A (*N* = 59 with valid fNIRS data) and sub-sample B (*N* = 62 with only valid ECITT data), participants had higher mean accuracy scores on prepotent trials (sub-sample A; *M* = 0.928, *SD* = 0.083, sub-sample B; *M* = 0.913, *SD* = 0.089) compared to inhibitory trials (sub-sample A; *M* = 0.545, *SD* = 0.294, sub-sample B; *M* = 0.561, *SD* = 0.284). To test whether accuracy significantly differed by trial type or between sub-samples, we conducted a 2 × 2 mixed ANOVA with trial type (inhibitory or prepotent) as the within-subjects factor and sub-sample (A or B) as the between-subjects factor. Results revealed a significant effect of trial type; *F* (1, 119) = 161.411, *p* < .001, *η*p^2^ = 0.576, confirming that infants are significantly more accurate on prepotent (*M* = 0.921, *SE* = 0.008) compared to inhibitory trials (*M* = 0.553, *SE* = 0.026). There was no significant effect of group: *F* (1, 119) = 0.000, *p* = .994, *η*p^2^ < 0.001, and no significant group by trial type interaction: *F* (1, 119) = 0.278, *p* = .599, *η*p^2^ =0.002.

### 3.2 fNIRS results

Repeated measures ANOVAs identified 41 channels (out of 42 included in analyses) that demonstrated a main effect of time, i.e., a significant (*p* <0.05) increase in HbO_2_ and/or decrease in HHb from base-line over time (see SM 7a, Supplementary Tables 11 and 12 for list of channels and associated statistics). A main effect of time means that the signal in these 41 channels changed significantly from the baseline over the task block time course.

A total of 27 channels showed both a significant HbO_2_ increase and a significant HHb decrease from baseline over time. Six channels showed only a significant HbO_2_ increase from baseline over time and eight channels showed only a significant HHb decrease. After correction for the FDR (84 comparisons), all channels retained significance (except Channel 6, HbO_2_ only, which was then excluded from further analyses).

**Fig. 4:**
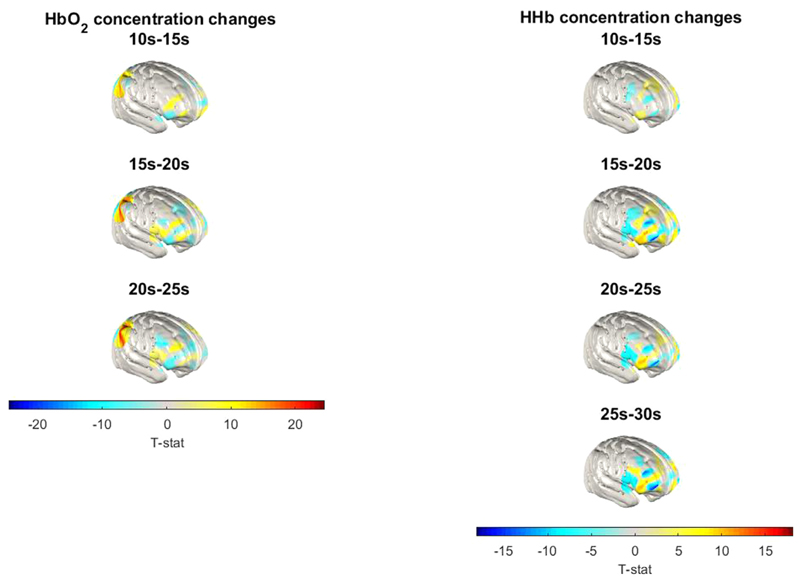
Group-level T-statistic Images of the Contrast in Concentration Changes between Block Types *Note*. Group-level T-statistic images of the contrast in concentration changes of each chromophore (HbO_2_ on the left and HHb on the right) between experimental and control blocks (experimental – control). Images are displayed in the space of a cortical surface derived from averaged structural MRI data of a 12-month-old cohort of infants (Shi et al., 2011). Using this approach, all displayed T-statistic values are significantly different across block types (paired *t*-test) at the alpha level of *p* < .01. The HbO_2_ figure (left) covers the 10 – 25 second time frame, and the HHb figure (right) covers the 10 – 30 second time frame with which our analyses in significant channels were conducted. Since we found no significant HHb effects in parietal regions, the HHb figure (right) only shows activation in frontal regions. A MATLAB version of this figure is available on OSF (https://osf.io/mv47n/).

Repeated measures analyses with the 40 channels showing robust HbO_2_/HHb changes over time (main effect of time) indicated six channels that also showed a significant main effect of block type (greater HbO_2_ increase/HHb decrease in experimental compared to control blocks), or a significant time × block type interaction, see Table 1. Three channels covering the right PFC (Channels 25, 26 and 32) and two covering the right parietal cortex (Channels 10 and 12) showed a significantly greater HbO_2_ increase in experimental compared to control blocks, and three channels overlaying the right PFC (Channels 25, 32 and 33) showed a significantly greater decrease in HHb in experimental compared to control blocks. See Fig. 4 for a *t*-statistic image of significant haemoglobin concentration differences between conditions (for visualisation purposes). Similar *t*-statistic images of significant haemoglobin concentration change by block type (compared to baseline) are presented in SM 7a (Supplementary Figures 5 and 6) and MATLAB versions of the *t*-statistic figures are available on OSF (https://osf.io/mv47n/).

#### 3.2.1 Channel localisation

From the localisation process (described in Section 2.6.5), pairs of channels that covered the same region of the brain were established. Two pairs of channels showing significant HbO_2_ effects were formed: Channels 10 and 12, overlaying the right parietal cortex, and Channels 25 and 26, covering the right PFC. Using the process described in Section 2.6.5, we were able to localise Channel 10 as covering the right inferior parietal gyrus and Channel 12 the right superior parietal gyrus; we will refer to this channel pair as the ‘right parietal cortex pair’. Channels 25 and 26 were localised to the right middle frontal gyrus, situated in the ventral region of the right DLPFC (Petrides and Pandya, 2012); the ‘right DLPFC pair’. The pair of channels showing significant HHb effects (Channels 32 and 33) was localised to the orbital middle frontal gyrus; the ‘orbital right PFC pair’. For the time course analysis, we decided not to include the HbO_2_ data from Channel 32 in the frontal pair (with Channels 25 and 26) because Channel 32 is in a more anterior part of the right PFC (specifically, the orbital PFC) whereas Channels 25 and 26 overlay the dorsolateral PFC. Therefore, we consider Channel 32 independently for HbO_2_ and as part of the right orbital PFC pair for HHb. We justify this decision in full detail in SM 7b and in the pre-registration (https://osf.io/qs4h8/).

#### 3.2.2 Time course of block type effect

For each individual channel or channel pair that was identified in our initial analyses as showing a significant block type effect or interaction (described above and see Table 1), paired *t*-tests were conducted to investigate the time course of the effect. Results are reported in Table 2. Note that for Channel 32 (HbO_2_), the significant effect occurred in a time-bin where we would expect activation to still be building at the start of the block, and the difference was in the opposite direction to what was observed for the other channels. Therefore, we did not further analyse or interpret the HbO_2_ data from Channel 32.

#### 3.3 Association between fNIRS data and individual differences in ECITT performance

In line with our pre-registration, we performed one-tailed correlational analyses to investigate potential positive associations between individual differences in infants’ inhibitory score and activation in channels overlaying the right lateral PFC, right parietal cortex, and right orbital PFC. The channels included in the individual differences analyses were previously identified in the group-level analyses (see Section 3.2. above) as showing significant block-type effects or interactions in either the HbO_2_ or HHb signal (see Table 1).

HbO_2_ difference data (described in Section 2.6.4 and in the ‘Indices’ section of the pre-registration) from Channels 25 and 26 were averaged, first *within* each individual time-bin (1 – 7), and then the averaged data from each time-bin was averaged *across* the pre-identified time-bins (in this case, bins 3 – 5) to form the variable ‘HbO_2_ right lateral PFC’. The same approach was taken to generate the variables ‘HbO_2_ right parietal’ (although with data from Channels 10 and 12) and ‘HHb right orbital PFC’ (with HHb difference data from Channels 32 and 33 from time-bins 4 – 6). A ‘HHb Channel 25′ variable was also generated in this way using HHb difference data from Channel 25 across time-bins 3 – 5. Note that whilst Channel 26 was included in averaging with Channel 25 in the variable ‘HbO_2_ right lateral PFC’ as described above, Channel 26 was not included in the ‘HHb right lateral PFC’ difference measure as there were no significant effects in the HHb signal in Channel 26.

**Fig. 5:**
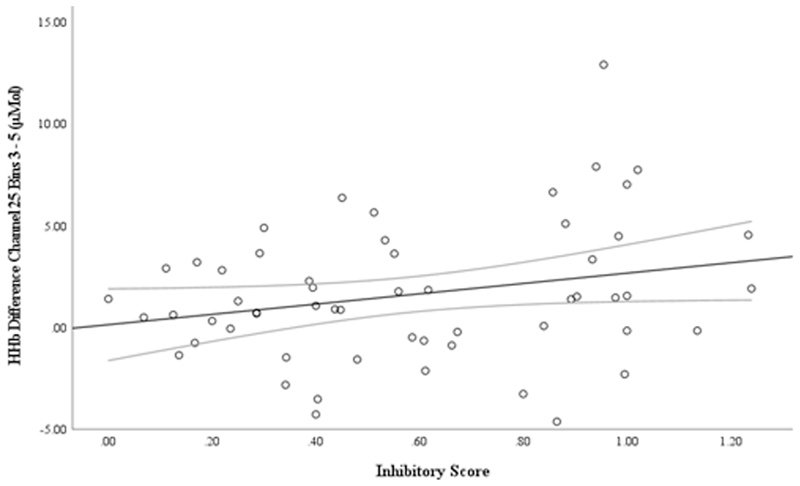
Association between the HHb difference score (Channel 25, Bins 3 – 5) and Inhibitory Score *Note*. A larger inhibitory score is indicative of better response inhibition performance on the ECITT. A larger HHb difference score reflects a larger HHb decrease in experimental compared to control blocks in Channel 25 overlaying the right PFC area.

There was no significant association between individual differences in infants’ inhibitory score and HbO_2_ activation in the right lateral PFC pair; *r* (57) = −0.056, *p* = .338, [*CI* = −0.287, 0.156], or in the right parietal pair; *r* (53) = 0.171, *p* = .106, [*CI* = −0.134, 0.423]. A modest, significant positive association was found between HHb concentration difference from 10 – 25 s in Channel 25 (right PFC) and inhibitory performance; *r* (54) = 0.255, *p* = .029, [*CI* = 0.016, 0.446], see Fig. 5. This suggests that the HHb concentration difference in Channel 25 across this time window is weakly associated with individual differences in infants’ response inhibition performance on the ECITT task in the predicted direction. However, since this effect did not survive correction for three tests this cannot be considered a robust result and must be replicated in future research.

#### 3.3.1 Exploratory analyses

We also pre-registered that we would perform exploratory analyses to examine whether the difference in HHb concentration across time-bins 4 – 6 (15 – 30 s of the block time course) in the right orbital PFC channel pair (Channels 32 and 33) was associated with infants’ response inhibition performance. Note that the broad label of ‘right anterior PFC’ was used in the pre-registration in reference to Channels 32 and 33, however these channels were later localised more specifically to the right orbital PFC.

Whilst there was no significant association between HHb concentration difference in the right orbital PFC and inhibitory performance; *r* (57) = −0.222, *p* = .092, [*CI* = −0.423, −0.043], we did find a significant association between HHb concentration difference independently in Channel 33 and inhibitory performance; *r* (57) = −0.273, *p* = .036, [*CI* = −0.476, −0.056]. The direction of the correlation suggests that greater HHb activation (a larger decrease) in experimental compared to control blocks is associated with *poorer* response inhibition performance (see SM 7d for correlation scatterplots). This effect is of a similar, although slightly stronger magnitude to that found at the pair-level, and so suggests that Channel 33 was likely driving this effect. As this result was exploratory, caution must be taken when interpreting these results and replication is required in future research.

## 4 Discussion

This study aimed to elucidate the neural correlates of response inhibition in 10-month-old infants. We did this by employing fNIRS, a technique that offers a unique insight into the localisation of activation in the awake infant brain, alongside a novel touchscreen task designed to elicit response inhibition in infants and toddlers (ECITT; Holmboe et al., 2021). We demonstrated that it is possible to successfully measure functional neural activation in infants who are engaged in an active task that requires a motoric response. Furthermore, our results, which align with existing research with pre-schoolers (Mehnert et al., 2013; Moriguchi and Shinohara, 2019; Yanaoka et al., 2020), older children (Cope et al., 2020; Zhou et al., 2022) and adults (see Aron et al., 2014 for review), provide new evidence for the involvement of right-lateralised regions of the prefrontal and parietal cortices when inhibitory control is exerted already during the first year of life.

### 4.1 Brain regions associated with response inhibition in 10-month-old infants

Our behavioural results confirmed that infants were significantly more accurate when responding to a target in a frequently rewarded location (prepotent trials) than in an alternative, less frequently rewarded location (inhibitory trials). This replicates previous findings in independent participant samples indicating that the ECITT is suitable as a measure of inhibitory control in infants (10 months; Hendry et al., 2021), toddlers (16–30 months; Hendry et al., 2021; Holmboe et al., 2021) and across the lifespan (4 years to adulthood; Holmboe et al., 2021).

From our fNIRS data, we identified six channels covering the right parietal cortex, right DLPFC and right orbital PFC that displayed significantly greater activation when inhibition was required. Our findings converge with previous work demonstrating activation in the right DLPFC during inhibitory control tasks across a wide age range, including early to middle childhood (Mehnert et al., 2013; Moriguchi and Shinohara, 2019; Yanaoka et al., 2020; Zhou et al., 2022), adolescence (Tamm et al., 2002), and adulthood (Aron et al., 2003, 2004; 2014). Our study also shows that, like older children and adults (Kolodny et al., 2020; Mehnert et al., 2013; van Belle et al., 2014), 10-month-old infants activate the right parietal cortex when inhibition is required. This is consistent with evidence that the prefrontal and parietal cortex work in conjunction during early EF development (Fiske and Holmboe, 2019). Finally, our results are further supported by a large longitudinal fMRI study of 7 – 23 year olds (*N* = 290) which found activation in the orbital frontal cortex, right DLPFC (Brodmann area 9/46), inferior frontal gyrus (ventrolateral PFC) and the superior parietal lobule during a Go/No-Go task (Cope et al., 2020). Although we did not find inhibition-specific activation in the ventrolateral PFC in the current study, it has been suggested that activation in this area during inhibitory control tasks increases progressively with age (Rubia et al., 2006).

Activation in channels covering the right orbital PFC, primarily in the HHb signal, was also found in the current study. Although there is limited evidence of orbitofrontal involvement in response inhibition in early childhood, previous neuroimaging studies with older children and adults have highlighted the involvement of this region in response inhibition tasks (Casey et al., 1997; Cope et al., 2020; Rubia et al., 2006), indicating that inhibition-specific activation in this area may increase with age. The results of the current study suggest that the right orbital PFC plays a role in inhibitory control from as early as 10-months of age. Future research is needed to replicate our finding and demonstrate age-related changes in right orbital PFC activation from infancy and across early childhood.

When assessing associations between fNIRS data and individual differences in behavioural performance on the ECITT, we found no evidence to suggest that individual differences in infants’ inhibitory performance were driven by the magnitude of the HbO_2_ response in the experimental (relative to control) blocks in channels over the frontal or parietal regions. However, we did find preliminary evidence indicating a weak association in the predicted direction between HHb activation in Channel 25 (overlaying the right DLPFC) and response inhibition, such that greater HHb activation was associated with better inhibitory performance. Since this association did not survive correction for the FDR, replication in future studies is required. We also found a weak association between HHb activation in Channel 33 (overlaying the right orbital PFC) and *poorer* response inhibition performance. However, this association was identified from exploratory correlational analyses and, as such, should be pre-registered and replicated in future research to confirm its validity. Nonetheless, these results provide new evidence for associations between individual differences in inhibitory control and neural activation in areas of the PFC in infancy; a preliminary but promising trend to be followed up in future research.

### 4.2 Interpretation of the fNIRS signal and brain-behaviour associations in infancy

The interpretation of fNIRS data from the current study hinges on the inherent differences between the two block types: the control block, which has no inhibitory demand but instead develops a prepotent response to one location, and the experimental block, which requires response inhibition to be exerted. We broadly interpret the condition effect found in channels covering the right DLPFC, orbital PFC and parietal cortex as being driven by the need for inhibition in experimental blocks. However, it is important to consider other potential explanations for the condition effect observed in channels covering these cortical regions.

Firstly, it could be that the brain activation found in this study was in response to infrequent events, rather than the need for inhibition. This is because inhibitory trials (where a response on the opposite side of the screen is required) occurred less frequently than prepotent trials. Indeed, previous fMRI research by Wijeakumar et al. (2015) highlighted that response control networks are modulated by both inhibition *and* rare events. However, in our study infants were presented with multiple experimental blocks and each of those had equal numbers of prepotent and inhibitory trials, so inhibitory trials, although still infrequent across the task (25% of trials), were not rare. Furthermore, if the haemodynamic response found in our data was driven by novelty or rare events, we would not have expected to observe the pattern of data we found, but rather a large increase at the beginning of the time course followed by a fast levelling out once inhibitory trials become more familiar.

Secondly, the timing of the reward (animated cartoon) after each trial may have influenced which areas of the cortex were more active in the experimental compared to the control condition. This is because after an incorrect response (which, by design, should be more frequent on inhibitory trials/in experimental blocks), the infant needs to change their response to the correct location to release the reward. It is possible that the activation in channels covering the right orbitofrontal cortex, a region of the brain that is rarely associated with response inhibition in young children (but see Casey et al., 1997), may reflect the processing of erroneous responses/perseveration and the associated lack of immediate reward (Happaney et al., 2004; Rolls, 2004) – perhaps more so for infants who struggle with the inhibitory demand of the task (and therefore make more errors). However, it seems unlikely that the activation observed in this study in channels covering the dorsolateral prefrontal and parietal areas is due to reward-related processing. This is because the DLPFC is commonly associated with ‘cool’ EF (no motivational or emotional involvement; Zelazo and Müller, 2011), whereas the orbitofrontal PFC is more typically associated with ‘hot’ EF and reward processing (Happaney et al., 2004; Zelazo and Carlson, 2012. Further, we found that increased activation in channels overlying the DLPFC area during experimental blocks was associated with *better* performance, thus suggesting that activation in the DLPFC is related, at least in part, to the inhibitory demands in the experimental block. Nonetheless, the influence of reward on neural activation during the ECITT is an interesting empirical question that needs to be addressed in future research.

Finally, it is important to acknowledge that it is somewhat difficult to interpret the brain-behaviour associations (or lack of) in our infant dataset. Since significant developments are occurring in the neurovascular system throughout infancy (Kozberg and Hillman, 2016), we would expect disparity and variability in the shape of the infant HRF as a result of age, brain region, and experimental design (Issard and Gervain, 2018; Lloyd-Fox et al., 2010). Similarly, cellular energy metabolism and its relation to haemodynamic changes in the infant brain are still not fully understood, meaning that any significant associations need to be interpreted carefully until more is known about these mechanisms (Siddiqui et al., 2017). It has also been argued that the infant brain does not ‘rest’ like the mature brain, bringing into question whether measurement of brain activity during baseline periods is truly indicative of rest in young participants, in the same way as adults (Camacho et al., 2020). With these issues in mind, we believe that the preliminary brain-behaviour associations found in this study, although weak, will be of interest to the field and warrant further investigation in future work.

### 4.3 Future directions

In this study, we investigated the haemodynamic response in channels overlaying specific regions of the brain and compared this across inhibitory and non-inhibitory conditions. The aim of this comparison approach was to identify brain regions that were linked to response inhibition in 10-month-old infants. It is unlikely that the brain regions identified in this study are working in isolation, and previous work has suggested that functional connections between frontal and parietal cortices strengthen with development to support the improvement of EFs (e.g., Buss and Spencer 2014, Buss and Spencer, 2018; see also Fiske and Holmboe, 2019). Although connectivity analyses are not compatible with our analysis approach and are therefore beyond the scope of this study, we propose that future research should examine functional connectivity between the regions of interest (e.g., channels covering the right PFC, right parietal cortex, and the right orbital PFC) to assess whether the interactions between brain areas are associated with infants’ inhibitory performance.

The multi-channel fNIRS probe used in the study allowed us to measure haemoglobin changes over a substantial area of the brain, sampling the bilateral prefrontal and parietal cortices that have previously been linked to response inhibition. Multi-channel fNIRS systems (as well as other multi-channel neuroimaging methods) face limitations in that there is an increased risk of finding false positive results due to running multiple parallel tests (Singh and Dan, 2006). When using the Benjamini-Hochberg procedure to control the false discovery rate in the analysis of our fNIRS data, the significant block type effects did not survive correction in all channels, although this was not unexpected due to the high number of comparisons (*N* = 84). Therefore, these results will require replication. Further studies that map the neural correlates of response inhibition across the infancy and early childhood period using a manual response task are needed to support the results of the current study.

Finally, we acknowledge that the level of data attrition in this study was substantial (~51%), although not uncommon in fNIRS studies with infant participants. According to a recent meta-analysis on attrition rate in infant fNIRS studies, the average attrition rate in the 10 included studies was 34.23% (Baek et al., 2021). Evidence from this meta-analysis revealed that older infants show less attrition than younger infants, although the studies included in this meta-analysis assessed infants from 0 – 2 years (Baek et al., 2021), a considerable age bracket. However, when examining infant fNIRS studies involving participants of a similar age to our current sample, we found that our attrition rate is comparable (e.g., Bulgarelli et al., 2019 (~76% at 11-months); Hakuno et al., 2020 (~63% at 10–12 months), Miguel et al., 2019 (45% at 12-months). Despite the attrition, a particular strength of our study is that our sample of participants with valid fNIRS data (*N* = 59) was substantially larger than typical sample sizes for infant fNIRS studies (e.g., de Klerk et al., 2019; Lloyd-Fox et al., 2015). Additionally, the thorough data quality control processes we incorporated into our exclusion criteria and pre-processing meant that the fNIRS data included in our final sample was of high quality (something that is not always achievable with noisy infant data). Future research that replicates our findings in a significantly larger sample of infant participants - particularly a more ethnically and socio-economically diverse group - would further strengthen the conclusions drawn from the current study.

## 5 Conclusions

This study provides new evidence that, already at 10 months of age, infants engage their prefrontal and parietal cortices while attempting to inhibit responses. Specifically, we established, in a large sample of infants, that channels covering regions of the right lateral PFC and parietal cortex are more active in task blocks that require response inhibition than in blocks where inhibition is not required. These findings are broadly consistent with existing fMRI/fNIRS studies with older children and adults but have not previously been established in children under 1 year of age. It is well known that the EF network, which the prefrontal and parietal cortices are part of, undergo significant maturation and across childhood (Fiske and Holmboe, 2019). However, with this study we demonstrate that, despite the potential immaturity, these areas are engaged during response inhibition even in the first year of life.

## Supplementary Material

Supplementary material associated with this article can be found, in the online version, at doi: 10.1016/j.neuroimage.2022.119241.

Supplementary material

## Figures and Tables

**Table 1:** Significant HbO_2_ and HHb concentration change differences between block types.

Channel	Location	Signal		Statistic
10	Right inferior parietal cortex	HbO_2_	Main effect of block type Time × block type interaction	*F* (1, 51) = 6.374, *p =* .015, *η*p^2^ = 0.111 *F* (3.307, 168.653) = 3.258, *p* = .019, *η*p^2^ = 0.060
12	Right superior parietal cortex	HbO_2_	Main effect of block type	*F* (1, 50) = 5.113, *p* = .028, *η*p^2^ = 0.093
25	Right middle frontal gyrus/DLPFC	HbO_2_	Main effect of block type Time × block type interaction	*F* (1, 55) = 4.139, *p* = .047, *η*p^2^ = 0.070 *F* (3.915, 215.306) = 3.172, *p* = .015, *η*p^2^ = 0.055
		HHb	Main effect of block type	*F* (1, 55) = 9.457, *p* = .003, *η*p^2^ = 0.147
26	Right middle frontal gyrus/DLPFC	HbO_2_	Time × block type interaction	*F* (3.907, 218.819) = 3.224, *p* = .014, *η*p^2^ = 0.054
32	Right orbital PFC	HbO_2_	Time × block type interaction	*F* (4.105, 229.852) = 3.874, *p* = .004, *η*p^2^ = 0.065
		HHb	Main effect of block type Time × block type interaction	*F* (1, 56) = 8.041, *p* = .006, *η*p^2^ = 0.126 *F* (3.923, 219.713) = 5.170, *p* = .001, *η*p^2^ = 0.085
33	Right orbital PFC	HHb	Time × block type interaction	*F* (2.850, 165.322) = 3.910, *p* = .011, *η*p^2^ = 0.063

*Note*. All 6 channels showed a significant main effect of time (*p* < .05), see SM 7a for statistics. None of the effects presented in this table survived correction for the FDR (84 comparisons).

**Table 2:** Time course of significant HbO_2_ or HHB block type effects.

Time Bin	Right parietal (HbO_2_)	Right DLPFC (HbO_2)_	Right orbital PFC (HbO_2_)	Right DLPFC (HHb)	Right orbital PFC (HHb)
0 – 5	*t* (47) = –0.687, *p* = .495, *d* = –0.099	*t* (53) = 1.082, *p* = .284, *d* = 0.147	***t* (56) = 2.379, *p* = .021, *d* = 0.315**	*t* (55) = 1.749, *p* = .086, *d* = 0.234	*t* (56) = –0.328, *p* = .744, *d* = –0.043
5 – 10	*t* (47) = –1.175, *p* = .246, *d* = –0.170	*t* (53) = –0.007, *p* = .994, *d* = –0.001	*t* (56) = 0.806, *p* = .424, *d* = 0.107	***t* (55) = 2.174, *p* = .034, *d* = 0.291**	*t* (56) = 0.509, *p* = .613, *d* = 0.067
10 – 15	*t* (47) = 2.485, *p* = .017, *d* = –0.359*	***t* (53) = –2.478, *p* = .016, *d* = 0.337**	*t* (56) = –1.489, *p* = .142, *d* = –0.197	*t* (55) = 2.898, *p* = .005, *d* = 0.387 *	*t* (56) = 2.802, *p* = .007, *d* = 0.371*
15 – 20	***t* (47) = –3.335, *p* = .002, *d* = –0.481**	***t* (53) = –2.055, *p* = .045, *d* = –0.280**	*t* (56) = –1.752, *p* = .085, *d* = –0.232	*t* (55) = 3.180, *p* = .002, *d* = 0.425*	*t* (56) = 2.771, *p* = .008, *d* = 0.367 *
20 – 25	*t* (47) = –3.541, *p* = .001, *d* = –0.511*	*t* (53) = –1.812, *p* = .076, *d* = –0.247	*t* (56) = –0.469, *p* = .641, *d* = –0.062	*t* (55) = 3.557, *p* = .001, *d* = 0.475*	*t* (56) = 3.104, *p* = .003, *d* = 0.411*
25 – 30	*t* (47) = –1.995, *p* = .052, *d* = –0.288	*t* (53) = –1.472, *p* = .147, *d* = –0.200	*t* (56) = 0.442, *p* = .660, *d* = 0.059	***t* (55) = 2.145, *p* = .036, *d* = 0.287**	***t* (56) = 3.049, *p* = .004, *d* = 0.404**
30 – 35	*t* (47) = –1.947, *p* = .058, *d* = –0.281	*t* (53) = –1.721, *p* = .091, *d* = –0.234	*t* (56) = –0.005, *p* = .996, *d* = –0.001	*t* (55) = 1.837, *p* = .072, *d* = 0.245	*t* (56) = 2.495, *p* = .016, *d* = 0.330*

*Note*. Statistically significant results are highlighted in bold.

Right parietal (HbO_2_) = Channels 10 and 12, Right DLPFC (HbO_2_) = Channels 25 and 26, Right orbital PFC (HbO_2_) = Channel 32, Right DLPFC (HHb) = Channel 25, Right orbital PFC (HHb) = Channels 32 and 33.

* significant after correcting the FDR (7 comparisons).

## Data Availability

The data that support the findings of this study and the custom MATLAB scripts used to analyse the fNIRS data is available on the Open Science Framework (OSF) website [https://osf.io/mv47n/] under a CC-By Attribution 4.0 International license (please cite this article if using any of these materials). The code for the original ECITT task and the blocked version of the ECITT used with fNIRS are available on Figshare [https://figshare.com/articles/software/ECITT_Web_App/13258814]. See Holmboe et al. (2021) for details on how to access demo versions of both tasks. The code used to analyse the fNIRS data (HomER2) is available at [https://www.nitrc.org/projects/homer2] and a MATLAB script of the pre-processing stream used in this study is available on OSF [https://osf.io/mv47n/]. The code used to produce the head model and reconstruct images has been developed and released via [http://www.github.com/DOT-HUB and [https://github.com/liamhywelcj/ReconstructionTenMonthCohortData]. The reconstructed images presented in this paper are also available as a MATLAB figure in the OSF project associated with this paper [https://osf.io/mv47n/].
