# Supplementary material for: The neural correlates of inhibitory control in 10-month-old infants: A functional near-infrared spectroscopy study
Source: Neuroimage. Author manuscript; Available in PMC 2024 Jul 31. (PMC7616317; doi:10.1016/j.neuroimage.2022.119241)
Supplement: Supplementary material [file EMS197764-supplement-Supplementary_material.docx]

The neural correlates of inhibitory control in 10-month-old infants: a functional near-infrared spectroscopy study

SUPPLEMENTARY MATERIALS

Abigail Fiske^*^, Carina de Klerk, Katie Y. K. Lui, Liam Collins-Jones, Alexandra Hendry, Isobel Greenhalgh, Anna Hall, Gaia Scerif, Henrik Dvergsdal, Karla Holmboe

^*^Corresponding author: Abigail Fiske, Department of Experimental Psychology, University of Oxford, United Kingdom. Email: abigail.fiske@psy.ox.ac.uk

*Note.* The following supplementary materials correspond to the article (as titled above) published in *NeuroImage* [<https://doi.org/10.1016/j.neuroimage.2022.119241>]. See also our project on the Open Science Framework [<https://osf.io/mv47n/>] for materials relating to the article, these supplementary materials, or the data to which these reports are associated. The materials in this project are under a CC-By Attribution 4.0 International license. Please cite the article if using any of these materials.

**Contents**

[Supplementary Materials 1 – The Oxford Early Executive Functions Study 3](#_Toc100660889)

[Supplementary Materials 2 – fNIRS 5](#_Toc100660890)

[2a - fNIRS Headgear Placement 5](#_Toc100660891)

[2b - fNIRS Channel Position 6](#_Toc100660892)

[2c - fNIRS Setup 7](#_Toc100660893)

[*Testing Setup* 7](#_Toc100660894)

[*Time Sync Protocol* 7](#_Toc100660895)

[2d – Short Separation Channels 8](#_Toc100660896)

[2e – Head Modelling 10](#_Toc100660897)

[**Supplementary Materials 3 - ECITT Coding Schemes 11**](#_Toc100660898)

[3a - Accuracy and Validity 11](#_Toc100660899)

[3b - Baseline Looking Behaviour 11](#_Toc100660900)

[Supplementary Materials 4 – fNIRS Data Exclusions & Processing 13](#_Toc100660901)

[4a - Block Exclusions 13](#_Toc100660902)

[4b – Data Processing in Homer2 14](#_Toc100660903)

[Supplementary Materials 5 – Behavioural version of ECITT (Session One) 16](#_Toc100660904)

[5a – Method and Results from ECITT Administered in Session One 16](#_Toc100660905)

[*Participants* 16](#_Toc100660906)

[*Stimuli* 17](#_Toc100660907)

[*Procedure* 17](#_Toc100660908)

[*Data Preparation and Analysis* 18](#_Toc100660909)

[*Results* 18](#_Toc100660910)

[5b - Test Re-test Reliability 20](#_Toc100660911)

[Supplementary Materials 6 – Blocked version of ECITT with fNIRS (Session Two) 22](#_Toc100660912)

[6a - Accuracy by Block Type 22](#_Toc100660913)

[6b - Parametric test assumptions 22](#_Toc100660914)

[6c – Behavioural Data; Results of Equivalent Non-Parametric Tests 23](#_Toc100660915)

[6d - Test Re-test Reliability (final fNIRS sample) 25](#_Toc100660916)

[6e – Reaction Time Data 26](#_Toc100660917)

[Supplementary Materials 7 – fNIRS Data 28](#_Toc100660918)

[7a - Group-level analyses 28](#_Toc100660919)

[7b - Justification for the exclusion of Channel 32 from the right PFC cluster (HbO_2_) 32](#_Toc100660920)

[7c - Individual Differences Analyses 33](#_Toc100660921)

[7d - Exploratory Analysis 34](#_Toc100660922)

[7e – Additional Group-Level Analysis (Including Time-Bins 8 and 9) 36](#_Toc100660923)

[References 39](#_Toc100660924)

# **Supplementary Materials 1 – The Oxford Early Executive Functions Study**

Participants were recruited from the Oxford University Babylab database to take part in a longitudinal study investigating the development of executive functions (EF) across the first three years of life. The study intended to follow 200 infants across four time points (10-, 16-, 24- and 30-months) using several behavioural (including eye-tracking), physiological (heart rate recording) and neural (electroencephalography and functional near-infrared spectroscopy) methods. Ten-month-old infants visited the laboratory for two testing sessions spaced approximately one week apart. Infants completed the Early Childhood Inhibitory Touchscreen Task (ECITT; Holmboe et al., 2021) in the first session, and a blocked version of the ECITT designed for use with functional near-infrared spectroscopy (fNIRS) in the second session (described in the article). The longitudinal study was discontinued in March 2020 because of the COVID-19 pandemic. Before the pandemic onset, 144 10-month-old infants participated in the first test session and 138 of these completed their second test session.

Participants were primarily White British and from families of middle to high socioeconomic status, as indicated by the mother’s years in education (*M* = 17.89 years). The ethnicity of this sample is reflective of the ethnic demographic of the city in which this research was conducted (according to the 2011 UK Census). Demographic information for the participants in this study (*N* = 135; 64 males) are detailed in Supplementary Table 1.

The longitudinal study inclusion criteria required that infants were born at full-term (at least 36 weeks) and/or with a minimum birth weight of 5.5 pounds or 2,500 g. All infants met these criteria. Infants with birth complications leading to health-related concerns were excluded from this sample (*n* = 3 males).

| Supplementary Table 1 | | | | | | | |
| --- | --- | --- | --- | --- | --- | --- | --- |
| *Full Sample Demographic Characteristics* | | | | | | | |
|  | *N* | | Mean | *SD* | Minimum | | Maximum |
| Infant’s age (days) at Session One | 135 | | 294.55 | 12.04 | 280.02 | | 308.93 |
| Infant’s age (days) at Session Two | 135 | | 313.01 | 6.22 | 301.00 | | 336.00 |
| Mother’s age | 134 | | 34.27 | 4.47 | 19 | | 47 |
| Mother’s years in education | 130 | | 17.89 | 3.00 | 11 | | 28 |
| Father’s age | 131 | | 35.66 | 5.19 | 22 | | 53 |
| Father’s years in education | 122 | | 17.27 | 3.35 | 9 | | 30 |
| Ethnicity | | *N* | | | | % | |
| Asian | | 3 | | | | 2.2 | |
| Mixed | | 12 | | | | 8.9 | |
| Other Ethnic Group | | 3 | | | | 2.2 | |
| Other White | | 20 | | | | 14.8 | |
| White British | | 95 | | | | 70.4 | |
| Prefer not to say/did not answer | | 2 | | | | 1.5 | |
| *Note.* Demographic information was collected via online questionnaire shortly before the child attended their first test session at 10-months. Some information was unavailable, as reflected in the N. | | | | | | | |

# **Supplementary Materials 2 – fNIRS**

## **2a - fNIRS Headgear Placement**

The validity of the fNIRS cap placement was assessed to ensure that the optical sensors that have been specifically organised to bilaterally cover the prefrontal cortex (PFC) and the area around the intraparietal sulcus were indeed covering these brain areas. In this way, we aimed to standardise across participants (as much as possible) the brain regions sampled. At the start of the test session, photographs or videos were taken as a record of how the fNIRS cap was positioned on the child’s head. If there was significant cap movement during the session, additional photographs/videos were recorded at the end of the session to document the movement of the cap on the head.

For all participants who the cap and completed at least three blocks of each condition (*n* = 99*),* two independent coders (AF & KL) viewed the photographs/video frames to assess the validity of the cap placement. Coders followed a simple ‘traffic-light’ style coding scheme (see Supplementary Table 2). Following this, coding discrepancies were reviewed by a third coder (KH) who made an independent judgement using the same coding scheme. The coders then agreed on a final decision which was in line with the coding of most coders. Based on this final coding, cap placement that was rated ‘good or ‘acceptable’ was deemed valid. Cap placement that was rated ‘poor’ was deemed invalid and the participant’s fNIRS data was excluded from further analyses (*n* = 19).

| Supplementary Table 2 | | |
| --- | --- | --- |
| *Cap Placement Coding Scheme* | | |
| Code | Rating | Description |
| Green | Good | The cap is sitting on the eyebrows or just very slightly above the eyebrows and looks straight on the head, with the central optode (FpZ) in the centre of the forehead, level with the eyebrows. |
| Amber | Acceptable | The cap is sitting a little high and/or a little lop-sided with the central optode (FpZ) slightly off-centre, but overall looks acceptable. |
| Red | Poor | The cap is sitting very high (half-way up the forehead or higher) and/or is not on straight at all, with the central optode being nowhere near the centre of the forehead (more than half-way over the eyebrow, from the middle of the eyebrow and beyond). |

## **2b - fNIRS Channel Position**

The position of each of the 46 channels included in the array are shown in Supplementary Figure 1 below.

| Supplementary Figure 1 |
| --- |
| *Channel Map* |
| *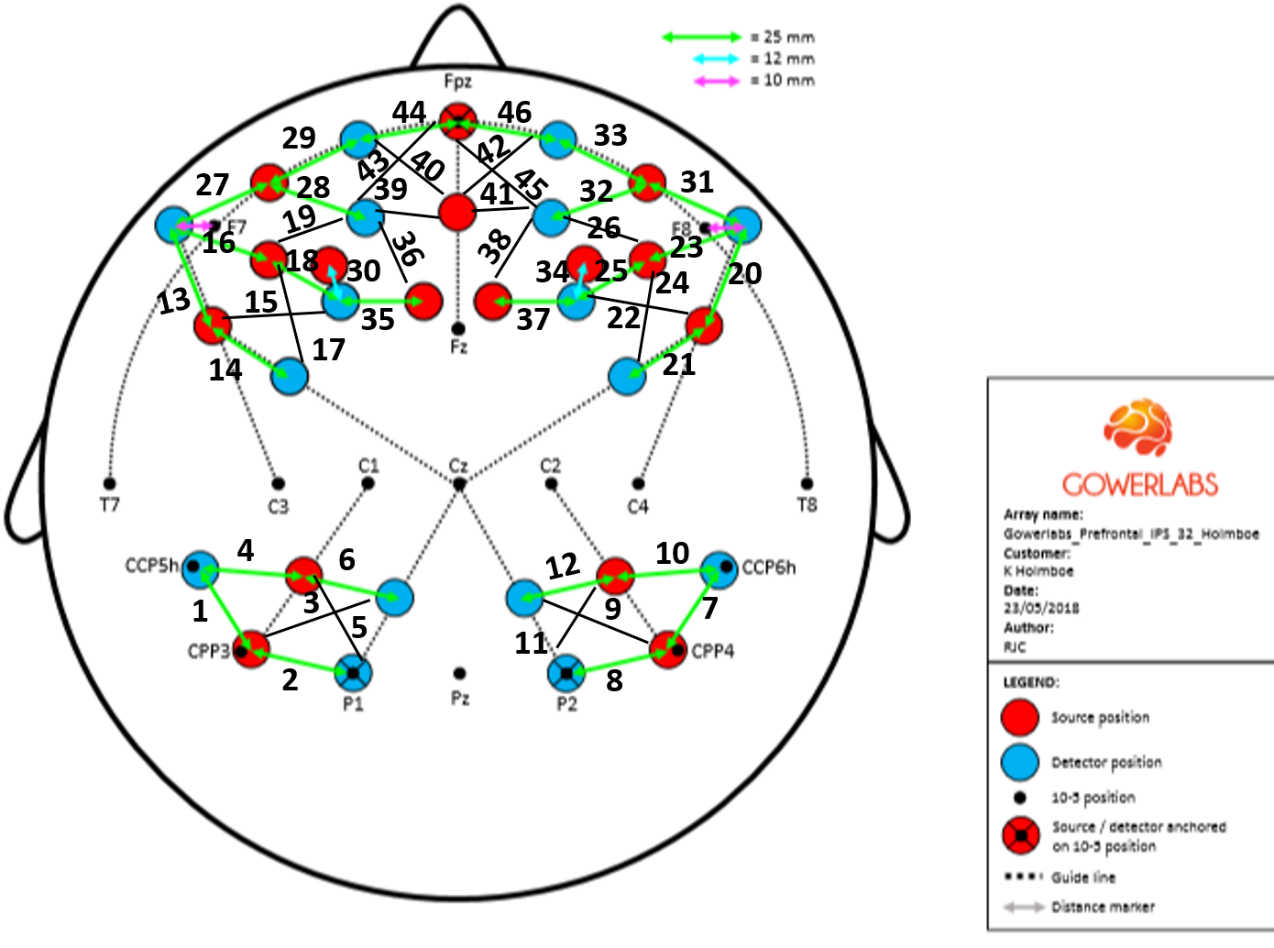* |
| *Note.* Sources are coloured red, detectors are blue. Black dots indicate the 10-5 position and dotted lines are guide lines. Optodes with crosses on are anchored onto 10-5 positions. |

## **2c - fNIRS Setup**

### *Testing Setup*

In our fNIRS setup, the NTS system is connected via USB to a Dell (Windows) laptop from which the fNIRS system is controlled (pictured on top of the NTS system in Supplementary Figure 2). We opted not to have the iPad directly connected to the NTS system as previous piloting indicated that anything on the iPad other than the screen is highly distracting for infants. Therefore, the NTS system was connected via USB to a Windows laptop on an adjacent bench (pictured to the right of the NTS system in Supplementary Figure 2). This second laptop was used to send timing information to the NTS system; see below for more details on the time sync process.

| Supplementary Figure 2 |
| --- |
| *fNIRS Testing Setup* |
| 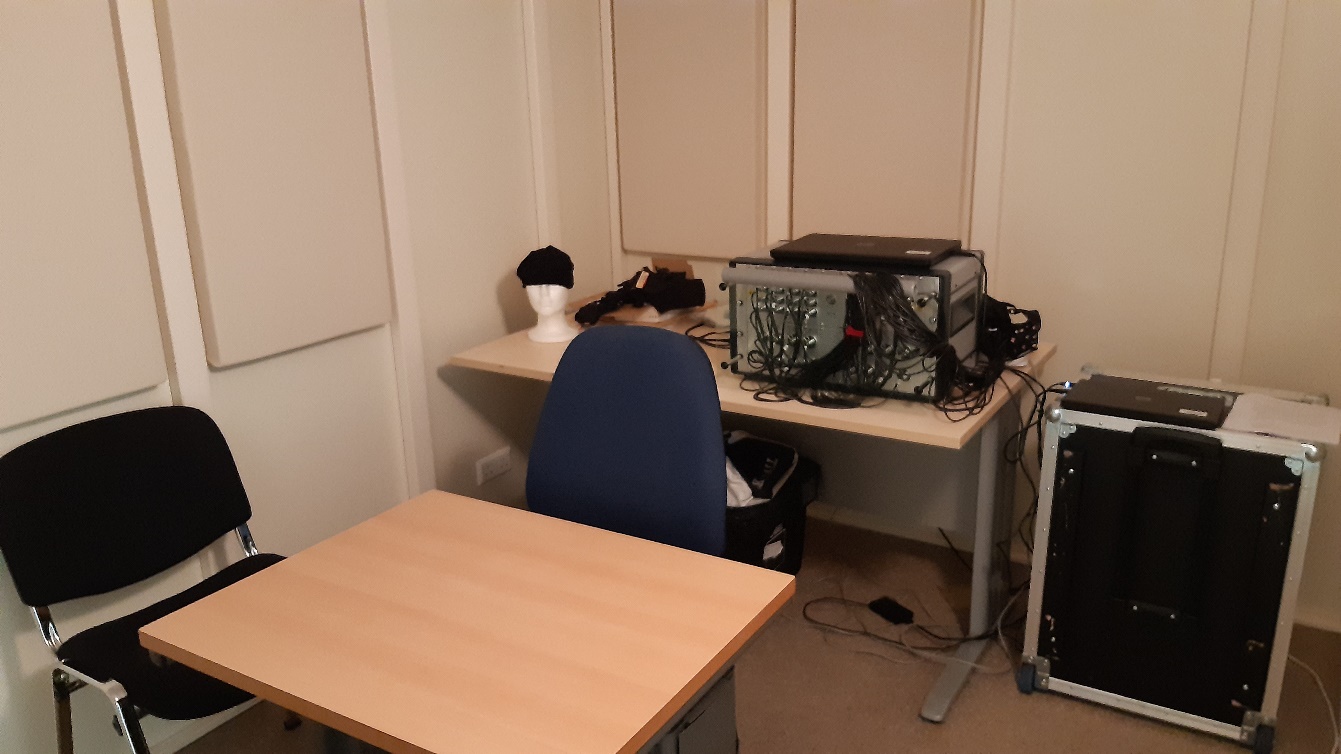 |

### *Time Sync Protocol*

The ‘sync laptop’ was used to run a custom MATLAB script (<https://osf.io/dexjn/>) which sent an exact time stamp to the data stream recorded by the NTS system. The time stamp was generated based on the internal laptop clock that was synced to the Apple server immediately before each test session. Because the fNIRS continuous data stream does not have an internal clock variable, we used this time stamp signal to allow us to align the event markers (collected through the ECITT software via the iPad) with the brain signal data collected by the fNIRS system. A separate script was used for this alignment which was done at the data processing stage; see below. The internal laptop clock used for the sync signal was synced to the Apple server immediately before each test session, whereas the internal iPad time was automatically synced to the Apple server (+/- 5ms) at least once a day.

We used a custom MATLAB script (available on <https://osf.io/3d4w2/>) to convert the universal output format (.txt) from the NTS system into .nirs format and to import event markers (start of each block) from the ECITT software, as well as the time sync information into the .nirs data. Discrepancies in time between that recorded by the ECITT software on the iPad and the time on the sync laptop were minimal in most instances (*M* = 252ms, *SD* = 365ms), although there were 4 cases in which the discrepancy was greater than 1 second (Maximum = 1556ms). Any discrepancy between the iPad time and the generated time-stamp marker (sync signal) will mean that there is a slight misalignment between the actual time the block started (e.g., when the first trial of a task block was presented) and the time at which the event marker is situated in the .nirs file. However, internal clocks in computers are inaccurate and so small random lags or discrepancies (typically on the scale of a few hundred milliseconds) per day are not uncommon. These random lags are unlikely to bias our data in any systematic way although they might add a little more noise when the delay is substantial. Given the slow temporal nature of the haemodynamic response, the rare cases of greater temporal misalignment would be expected to simply add a small amount of measurement error; this error would be the same across all block types, and therefore this slight timing disparity is very unlikely to induce any sort of spurious effect into our results.

## **2d – Short Separation Channels**

Our fNIRS probe was designed to include two shorter channels so that we could use short-separation regression to regress out physiological artifacts in the fNIRS signal. However, the short channels in our probe are positioned at a separation of 12 mm; a distance which is in fact not optimised for short separation regression techniques. According to Brigadoi & Cooper (2015), the optimum short separation distance is 8.4 mm for an adult head and 2.15 mm for a new-born infant, whereas Emberson et al., (2016) suggest a 1 cm (10mm) distance for a short channel with infant participants (~6 months). Practically, it is not possible to have channels this short due to the limitations of our fNIRS hardware.

The optimal distance for a short channel in the infant population is still disputed among researchers, particularly given the small scalp-to-brain distance in infants which makes it difficult to engineer a short channel with a sufficiently small separation to have negligible signal contribution from the brain. Zhou et al. (2020) discusses that in studies that have used longer source-detector separations to form their ‘short’ channels (i.e., 13 – 25 mm), there is a risk of regressing out or attenuating actual brain responses from the signal and thus reducing the signal to noise quality. Further, Emberson et al. (2016) found that whilst short-separation channels (1cm) were successful in removing non-cortical vascular signals from infant fNIRS data, this technique also reduced the signal to noise ratio. The authors also found that there was no overall effect of short separation techniques on the statistical inferences that could be drawn from the study. Finally, like Frijia et al. (2021), we also observed evidence of task-related brain activation when examining the HRFs in our short channels which suggests that the short-separation channels are not behaving as intended and, as such, it would not be appropriate to perform short-separation regression techniques.

The lack of standardised approaches for using short separation regression techniques with infant data (as mentioned in Frijia et al., 2021), as well as the fact that our short separation channels were arguably not short enough, means that it was not feasible (or indeed necessary) to conduct short separation regression on this dataset. Instead, to address physiological noise in our signal, we performed bandpass filtering (high pass, 0.01, and low pass, 0.80) which allowed us to remove some level of systemic noise from our data. Importantly, since we found differential activation in channels across the scalp, not just a global increase or decrease, indicating that our condition-effects are unlikely to be driven by physiological noise.

## **2e – Head Modelling**

To allow us to visualise the data on an age-appropriate head template, a model of the infant head was produced from averaged structural MRI data of a 12-month-old cohort (Shi et al., 2011). Group-level tissue masks were combined to produce a mask of the spatial distribution of the cerebral tissues (white matter, grey matter, and cerebrospinal fluid). The inner skull border was delineated by the outside boundary of the cerebral tissue mask, while the scalp surface was defined using the Betsurf procedure (Jenkinson et al., 2005) where the group-level T1-weighted MRI template was used as an input. All voxels situated between the inner skull border and the scalp surface were assigned to be extra-cerebral tissue; this is a combined label for scalp and skull, and is commonly done in infant head models due to the difficulty in discerning these two tissues in infant MRI data (Brigadoi et al., 2019; Collins-Jones et al., 2021; Frijia et al., 2021). The resulting four-layer tissue mask (consisting of white matter, grey matter, cerebrospinal fluid and extra-cerebral tissue) was converted to a tetrahedral volume mesh and a grey matter surface mesh using the iso2mesh package; Fang & Boas, 2009, see iso2mesh.sourceforge.net).

## **Supplementary Materials 3 - ECITT Coding Schemes**

### **3a - Accuracy and Validity**

Accuracy and validity of ECITT trials were coded offline from videos recorded during the test session. This coding was conducted in Excel, using output from the ECITT software recorded during the testing session (template available at <https://osf.io/fhnxp/>). The same coding scheme was used to code the ECITT (Session One) and the blocked version of the ECITT used with fNIRS (Session Two). The full details of the coding scheme are available on OSF (<https://osf.io/pkzxa/>).

### **3b - Baseline Looking Behaviour**

Infants’ behaviour during the baselines in the blocked version of ECITT used with fNIRS (Session 2) were coded offline in [The Observer XT](https://www.noldus.com/observer-xt?utm_term=the%20observer%20xt&utm_campaign=OTM%7CHuman%7CObserver+XT%7CBranded&utm_source=adwords&utm_medium=ppc&hsa_acc=5401040478&hsa_cam=1016207978&hsa_grp=54066166710&hsa_ad=430326945791&hsa_src=g&hsa_tgt=kwd-542729219969&hsa_kw=the%20observer%20xt&hsa_mt=e&hsa_net=adwords&hsa_ver=3&gclid=CjwKCAiAh_GNBhAHEiwAjOh3ZMMR5UkCcNbogNJQJnyQZ2XxZaT6TTl4s3BvKNKOar4_Z9T9z4oJsRoCe_EQAvD_BwE) (versions 14 and 15) by two trained coders (AF and KL). The onset and offset of baseline, control, and experimental blocks were marked to allow for durations to be calculated. ECITT trial responses in control and experimental blocks had already been coded in Excel using the coding scheme described above. The infants’ looking behaviour during baselines were coded to determine the validity of the baseline (see Supplementary Table 3). The baseline was considered as valid if the infant was looking at the screen, or somewhere neutral, for at least 60% of the baseline duration (baselines varied in duration from 12-17 seconds). Baselines were coded as invalid if the infant was not looking at the screen or somewhere else neutral for at least 60% of the baseline duration. Excellent inter-coder reliability was established across 41 videos (1,715 coding incidences): κ = .85. The baseline coding protocol is available on OSF (<https://osf.io/u8byz/>).

| Supplementary Table 3 | |
| --- | --- |
| *Baseline Looking Behaviours Coding Scheme* | |
| Behaviour | Description |
| Screen/neutral | The infant is either looking at the screen or is turning their *head*to somewhere neutral (the table, the walls on both sides, the floor etc.). This is valid looking behaviour. |
| Faces | The infant is looking at the experimenter’s face or turns to look at parent’s face. This does not contribute to valid looking behaviour as faces may evoke confounding neural activation. |
| Other (e.g., crying, turning around, fussing) | Use this code when the infant is: crying, fussing, turning their *body*to the left or right side (big movements), turning into parent (but not looking at their face), turning behind to look at the fNIRS system/computer or the second experimenter (but as we cannot see what the infant is looking at, we should not code that as faces), and when the infant takes off the NIRS cap/pulls out optodes during the baseline. This does not contribute to valid looking behaviour as is likely to introduce motion artifacts and noise into the fNIRS signal. |

# **Supplementary Materials 4 – fNIRS Data Exclusions & Processing**

## **4a - Block Exclusions**

Invalid blocks of trials were manually excluded from processing in HomER2 using the ‘stim toggle on/off’ functionality (Huppert, Diamond, Francheschini & Boas, 2009). Blocks were identified as invalid during video coding if they were:

- Not fully completed by the participant (e.g., the experimenter or parent completed one or more trials, or the participant stopped responding)
- The participant was excessively crying, fussing, or moving
- Issues with headgear (e.g., infant pulling out optodes)
- Technical difficulties with the iPad, internet connection, or ECITT app
- If the block duration was extra-long. This is defined (separately for each type; control or experimental) as the group mean duration + 2 standard deviations (see Supplementary Table 4).

As a result, data from 13 participants were excluded from further analyses (*n* = 8 due to the exclusion of invalid blocks and *n* = 5 due to the exclusion of extra- long blocks, resulting in the participants no longer having at least three valid blocks of each type).

A note on the decision to exclude extra-long blocks

We decided to exclude extra-long blocks from analysis because when participants take this length of time to respond, it is often due to boredom or a distraction that causes the child to disengage with the task. During this time, the brain signal is unlikely to be related to the task and thus noise is introduced into the data. However, since we are keen to capture individual differences in task performance and brain activation, it was important that we did not exclude those longer blocks of trials in which infants are responding to the task in a valid way, but generally have a slower response time. Therefore, we accepted the mean + 2 standard deviations (SD) as a cut-off value for excluding extra-long blocks.

| Supplementary Table 4 | | | | | | |
| --- | --- | --- | --- | --- | --- | --- |
| *Descriptive Statistics of Block Durations in the Blocked Version of the ECITT (seconds)* | | | | | | |
|  | Mean | *SD* | 2*SD* | Cut off  (Mean + 2*SD*) | Minimum | Maximum |
| Control | 43 | 17 | 34 | 77 | 17 | 158 |
| Experimental | 49 | 18 | 36 | 85 | 23 | 138 |

*Note.* The duration of each block for each participant with valid cap placement (N = 80) was calculated using The Observer XT software. Invalid blocks (first four reasons bullet-listed above) were removed before the calculation took place. Block duration information for each individual block (by participant) is available at: <https://osf.io/atg5f/>.

## **4b – Data Processing in Homer2**

Data were pre-processed in HomER2 (Huppert, Diamond, Francheschini & Boas, 2009). The processing functions and parameters we used in this study (as described in the article) are reported in Supplementary Table 5. A MATLAB script and .cfg file of this processing stream are available on OSF (<https://osf.io/mv47n/>).

| Supplementary Table 5 | | |
| --- | --- | --- |
| *Homer2 Pre-Processing Stream Parameters* | | |
| hmrIntensity2OD |  |  |
| enPruneChannels | dRange | 1e-03 1e+03 |
|  | SNRthresh | 2 |
|  | SDrange | 0.0 45.0 |
|  | reset | 0 |
| hmrMotionArtifactByChannel | tMotion | 1.0 |
|  | tMask | 1.0 |
|  | STDEVthresh | 15.5 |
|  | AMPthresh | 0.40 |
| hmrMotionCorrectSpline | p | 0.99 |
|  | turnon | 1 |
| hmrMotionCorrectWavelet | iqr | 1.5 |
|  | turn_on | 1 |
| hmrMotionArtifactByChannel | tMotion | 1.0 |
|  | tMask | 1.0 |
|  | STDEVthresh | 15.5 |
|  | AMPthresh | 0.40 |
| hmrBandpassFilt | hpf | 0.010 |
|  | lpf | 0.80 |
| hmrOD2Conc | ppf | 5.2 4.8 |
| enStimIncData_varagin | 1 | 0.0 0.0 |
| hmrBlockAvg | trange | -2.0 45.0 |

# **Supplementary Materials 5 – Behavioural version of ECITT (Session One)**

## **5a – Method and Results from ECITT Administered in Session One**

### *Participants*

Participants were 144 10-month-old infants (73 males), however in accordance with the longitudinal study’s exclusion criteria (described in SM 1), three male participants were excluded from analyses. Of these, 128 infants contributed valid ECITT data (exclusion criteria described in *Data Preparation and Analysis* section below). See Supplementary Table 6 for demographic characteristics of the full sample (N = 141).

| Supplementary Table 6 | | | | | |
| --- | --- | --- | --- | --- | --- |
| *Demographic Characteristics of Participants* | | | | | |
|  | *N* | Mean | *SD* | Minimum | Maximum |
| Infant’s age (days) at Session One | 141 | 294.00 | 5.88 | 294.00 | 319.00 |
| Mother’s age | 139 | 34.15 | 4.44 | 19 | 47 |
| Mother’s years in education | 135 | 17.73 | 3.13 | 10 | 28 |
| Father’s age | 136 | 35.73 | 5.34 | 22 | 53 |
| Father’s years in education | 127 | 17.22 | 3.34 | 9 | 30 |
| Ethnicity | *N* | % | | | |
| White British | 97 | 68.79 | | | |
| Other White | 21 | 14.89 | | | |
| Asian | 3 | 2.13 | | | |
| Mixed | 14 | 9.93 | | | |
| Other ethnicity | 4 | 2.84 | | | |
| Prefer not to answer | 2 | 1.42 | | | |

*Note*. Infant age is recorded in days, parent’s age and education are in years. Demographic information about the parents of some participants were unavailable, as reflected in the N.

### *Stimuli*

Participants completed the ECITT task (Holmboe et al., 2021) as a measure of behavioural response inhibition (fNIRS was not used with this task in Session One). In this task, infants are presented with two blue ‘buttons’ on the left and the right of a touchscreen and are required to touch the button that contains the target stimulus (a smiley-face icon) to receive an animated reward (4 seconds). The ECITT consisted of 32 experimental trials, of which the target stimuli appeared on the prepotent side of the screen in 75% of trials, and on the inhibitory location in 25% of trials. For at least the first three trials, the target would appear on the prepotent side so that the infant would develop the prepotent response to this side of the screen. The location of the target on subsequent trials was randomised with the constraints that the target could not appear on the prepotent side for more than five trials in a row, or on the inhibitory side for more than two trials in a row. See Holmboe et al. (2021) for full details as the task used in this study closely follow this design.

### *Procedure*

Prior to the first testing session, infants were assigned a prepotent location - either the left or right of the screen - which corresponded to the version of ECITT the participant was presented. This was counterbalanced across participants. If infants responded incorrectly on the first experimental trial, the experimenter changed the prepotent side and re-started the task. During the task, infants were seated on the lap of their caregiver and positioned at a table so that they could easily reach the experimental apparatus. The experimenter first demonstrated the task to the participant, and then the infant completed at least two practice trials. In the demonstration and practice trials, only one ‘button’ (containing the target stimuli) appeared on the screen. On the first experimental trial, the experimenter pointed to the target whilst instructing the child to “touch the happy face”. Where necessary, participants received verbal encouragement to keep them engaged and motivated to respond to the task, however the task was stopped early if the infant became noticeably distressed and/or stopped responding.

### *Data Preparation and Analysis*

Procedures for data preparation and analysis for the Session 1 ECITT data are identical to that described in the main article. Three coders were responsible for coding the ECITT task and a subset of 17 videos (534 trials) were independently coded to establish inter-rater reliability using Fleiss’ kappa (*κ* = .96 for accuracy; *κ* = .89 for validity). Data was excluded from analyses if the participant completed less than 16 trials (including at least two valid inhibitory trials, *n* = 9) or if an accuracy score of less than 60% on prepotent trials was obtained (*n* = 4). As a result, the final sample with valid data consisted of 128 participants.

#### Parametric Test Assumptions

The ‘prepotent accuracy’ and ‘inhibitory accuracy’ variables from the ECITT in Session One were not normally distributed (Shapiro-Wilk test; highest *p*-value = .012, lowest *p*-value = <.001, inhibitory skew; *z* = -.377, prepotent skew; *z* = -3.77). According to Kim (2013), the inhibitory skew is of an acceptable level, however the level of skew in the prepotent variable exceeds that of an acceptable level. Given the task design, we anticipated a high level of accuracy on prepotent trials and thus negative skew was expected on this trial type. The ‘adjusted AccD’ variable was also not normally distributed (Shapiro-Wilk test; *p* = .005) but the negative skew (*z* = - .304) was of an acceptable level (Kim, 2013). Since these variables were not normally distributed, results of parametric and the equivalent non-parametric tests are also reported in ‘Results’ below. Supplementary Figure 3 displays the distribution of mean accuracy scores on prepotent and inhibitory trials and of the adjusted AccD score.

### *Results*

Analyses were conducted in SPSS version 27, with three dependent variables: prepotent and inhibitory accuracy, and the adjusted accuracy difference score (adjusted AccD). Descriptive statistics (reported in Supplementary Table 7) revealed that infant’s accuracy was higher on prepotent trials than inhibitory trials. Results of a paired-samples *t*-test confirmed a significant difference between accuracy on prepotent and inhibitory trials: *t* (127) = -12.908, *p* <.001, *d* = -1.141. This result is in convergence with the related-samples Wilcoxon signed rank test; *z* = 8.779, *p* < .001.

| Supplementary Table 7 | | | | |
| --- | --- | --- | --- | --- |
| *Descriptive Statistics for the ECITT (Session One) Accuracy Measures; N = 128* | | | | |
|  | Mean | *SD* | Minimum | Maximum |
| Prepotent Accuracy | .879 | .110 | .611 | 1 |
| Inhibitory Accuracy | .515 | .308 | 0 | 1 |
| Adjusted AccD | .408 | .357 | -.55 | 1 |

| **Supplementary Figure 3** |
| --- |
| *Distribution of the Adjusted AccD score and Mean Accuracy Scores on Prepotent and Inhibitory Trials* |
| 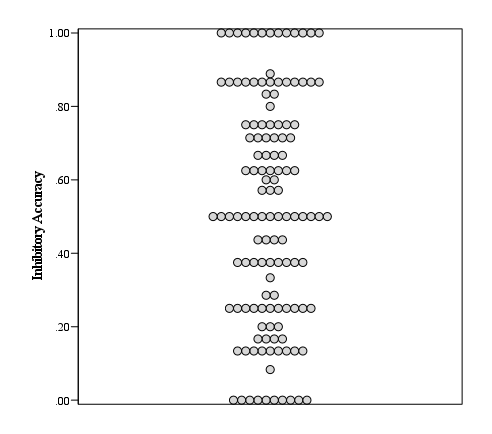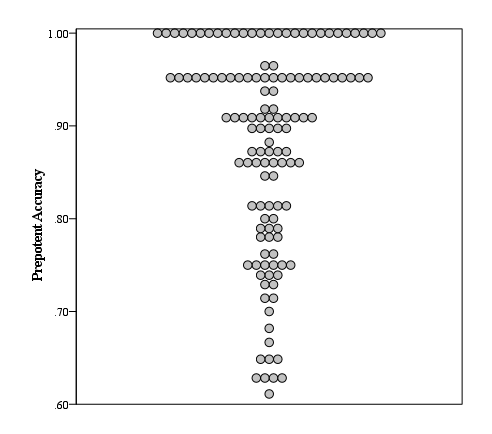  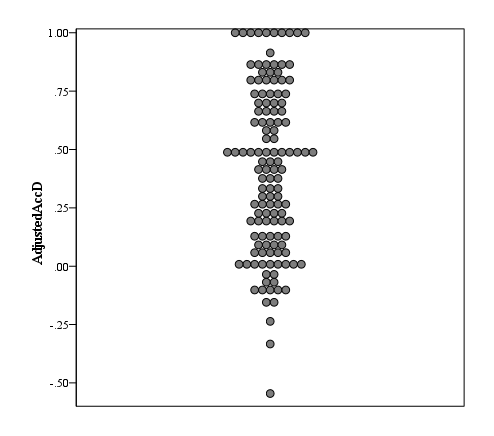 |

## **5b - Test Re-test Reliability**

Performance of the 112 infants who had valid ECITT data from both sessions (behavioural version of ECITT in Session One, and the blocked ECITT with fNIRS in Session Two) were compared. Results from the full sample mirror the results found in the sub-sample of 59 infants (the sample with valid fNIRS data from Session Two), which are reported in SM 6d. Pearson correlation coefficients revealed significant associations between ECITT performance in Session One and Session Two: adjusted AccD; *r* (110) = .482, *p* <.001, [*CI* = .296, .638], prepotent accuracy; *r* (110) = .321, *p* <.001, [*CI* = .081, .526], inhibitory accuracy; *r* (110) = .543, *p* <.001, [*CI* = .384, .669]. These results converge with the results of the non-parametric Spearman’s rho correlation tests: adjusted AccD; *r*_s_ (110) = .493, *p* <.001, [*CI* = .320, .640], prepotent accuracy; *r*_s_ (110) = .273, *p* = .004, [*CI* = .079, .464] and inhibitory accuracy; *r*_s_ (110) = .534, *p* <.001, [*CI* = .371, .675].

To confirm that there were no significant differences in performance between sessions, paired *t*-tests were conducted (see Supplementary Table 8 for descriptive statistics). Results revealed no significant differences in the adjusted AccD score; *t* (111) = .073, *p* = .942, *d* = .007 or inhibitory accuracy: *t* (111) = .941, *p* = .349, *d* = -.089 across the sessions. These results were in convergence with the non-parametric related-samples Wilcoxon signed rank test; adjusted AccD; *z* = .373, *p* = .709 and inhibitory accuracy; *z* = 1.237, *p* = .216. However, a significant difference in prepotent accuracy was observed: *t* (111) = -4.017, *p* <.001, *d* = -.380, indicating that accuracy on prepotent trials significantly improved from Session one to Session two. Again, this result is consistent with that from the non-parametric related-samples Wilcoxon signed rank test; *z* = 4.230, *p* < .001.

| Supplementary Table 8 | | | | | |
| --- | --- | --- | --- | --- | --- |
| *Descriptive Statistics for ECITT Performance across both Sessions; N = 112* | | | | | |
|  | Session One | |  | Session Two (fNIRS) | |
|  | Mean | *SD* |  | Mean | *SD* |
| Prepotent Accuracy | .883 | .109 |  | .925 | .082 |
| Inhibitory Accuracy | .524 | .303 |  | .550 | .291 |
| Adjusted AccD | .398 | .356 |  | .395 | .334 |

# **Supplementary Materials 6 – Blocked version of ECITT with fNIRS (Session Two)**

### **6a - Accuracy by Block Type**

Infants’ accuracy on prepotent and inhibitory trials is presented by block type (control and experimental) in Supplementary Table 9 below. All infants scored > 60% accuracy on prepotent trials across both block types.

| Supplementary Table 9 | | | | |
| --- | --- | --- | --- | --- |
| *Distribution of Mean Accuracy Scores (by Block Type) on Prepotent and Inhibitory Trials* | | | | |
|  | Control Blocks | | Experimental Blocks | |
|  | Mean | *SD* | Mean | *SD* |
| Prepotent Accuracy | .890 | .119 | .833 | .161 |
| Inhibitory Accuracy | - | - | .527 | .266 |
| *Note.* There were no inhibitory trials in control blocks. Data pertain to the full sample of participants with valid ECITT data in Session Two; *N* = 121. | | | | |

### **6b - Parametric test assumptions**

Supplementary Figure 4 (page 25 - 26) displays the distribution of mean accuracy scores on prepotent and inhibitory trials on the ECITT for infants in sub-sample A (N = 59, who also had valid fNIRS data) and sub-sample B (N = 62, who only had valid ECITT data). Participants were split into sub-samples so that we could establish whether there were any significant performance differences between infants with, and without, valid fNIRS data. The ‘prepotent accuracy’ and ‘inhibitory accuracy’ variables for both sub-samples were not normally distributed, according to the Shapiro-Wilk test (highest *p*-value = .016, lowest *p*-value = <.001). The z-scored skew for the inhibitory accuracy variable in both sub-samples was deemed as acceptable (*z* = .020 and -0.48), according to Kim (2013). For samples with more than 50 participants, a z-scored skew > 3.29 would suggest the sample distribution is not normal (Kim, 2013). The prepotent accuracy data were negatively skewed (*z* = -4.28 and -4.07), however, given the task design, we expected a high level of accuracy on prepotent trials in most participants due to the repetitive nature of the prepotent trials which elicits a strong response prepotency. As such, skew was expected on this trial type. The ‘adjusted AccD’ variable was also not normally distributed (Shapiro-Wilk test; *p* = .006) but the positive skew (*z* = .527) was of an acceptable level (Kim, 2013).

Whilst these variables met the necessary homogeneity of variance assumptions (Levene’s test; lowest *p*-value = .465, Box’s test; *p* = .941), the normality assumption was violated. Therefore, to guard against the assumption of normality we also ran the associated non-parametric tests for each parametric test we conducted using these variables.

### **6c – Behavioural Data; Results of Equivalent Non-Parametric Tests**

Results of a related-samples Wilcoxon signed rank test confirmed that the mean accuracy on prepotent and inhibitory trials was significantly different; *z* = 8.570, *p* <.001. Results of a Mann-Whitney U independent samples test confirmed that there was no significant difference in prepotent (*U* = 1609.000, *p* = .251) or inhibitory (*U* = 1870.500, *p* = .829) accuracy between sub-samples. These results are in convergence with the results of the parametric tests reported in the ‘Results’ section of the article.

| Supplementary Figure 4 |
| --- |
| *Distribution of Mean Accuracy Scores (by Sub-Sample) on Prepotent and Inhibitory Trials and the Adjusted AccD Score* |
| 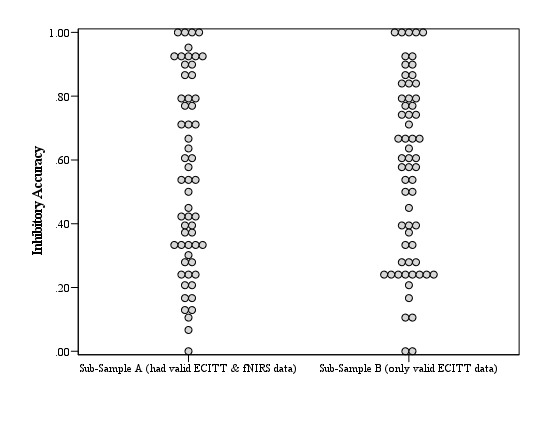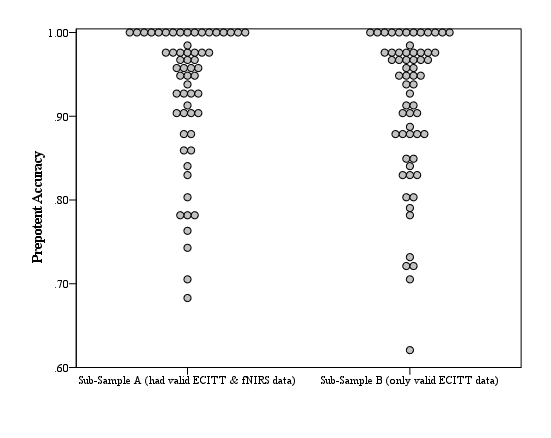  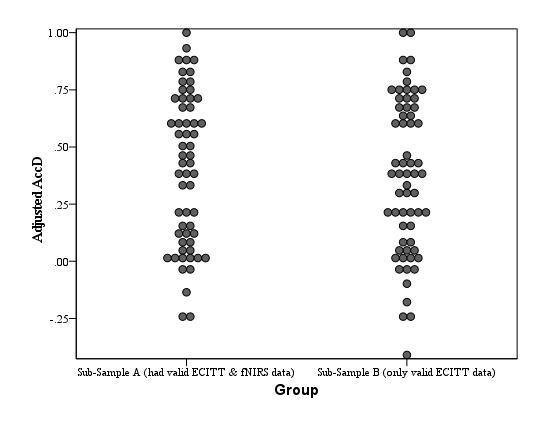 |

###

### **6d - Test Re-test Reliability (final fNIRS sample)**

Of the 59 participants with valid fNIRS and ECITT data, 57 infants also had contributed valid ECITT data during the first testing session (*n = 2* were excluded due to scoring <60% on prepotent trials). For these participants, ECITT data from the two testing sessions were compared to establish the test re-test reliability of the task.

Significant Pearson’s correlations indicated that performance on prepotent and inhibitory trials were moderately to strongly stable across sessions (Supplementary Table 10). Paired *t*-tests revealed no significant differences in inhibitory accuracy across the two sessions, although a significant difference in prepotent accuracy was observed, suggesting that infant’s accuracy on prepotent trials significantly improved from Session 1 to Session 2. The results of equivalent non-parametric tests (reported in Supplementary Table 10) were consistent with the results from the parametric tests, with one exception: the correlation in prepotent accuracy scores across test sessions was no longer significant when using Spearman’s rho. This suggests that performance on prepotent trials was not consistent from Session One to Session Two, as is also reflected in the significant paired *t*-test and Wilcoxon signed rank tests, which suggested that accuracy on prepotent trials was significantly higher in Session Two than in Session One (Supplementary Table 10).

| Supplementary Table 10  *ECITT Performance across Sessions (N = 57 infants with both valid ECITT and fNIRS data in Session Two, and valid ECITT data in Session One).* | | | | | | |  |
| --- | --- | --- | --- | --- | --- | --- | --- |
|  |  |  |  |  |  |  |  |
|  | ECITT (Session One) | ECITT-fNIRS (Session Two) | | Performance across sessions | | | |
|  | Mean (SD) | Mean (SD) | Paired *t*-test | | Wilcoxon signed rank test | Pearson’s Correlation | Spearman’s rho Correlation |
| Prepotent Accuracy | .892 (.106) | .935 (.076) | | *t* (56) = -3.106, *p* = .003^**^, *d* = -.411 | Z = 3.015, *p* = .003^**^ | *r* (55) = .383, *p* = .003^**^ [.052, .664] | r_s_ (55) = .229, *p* = .087 [-.069, .478] |
| Inhibitory Accuracy | .516 (.311) | .537 (.296) | | *t* (56) = -.590, *p* = .558, *d* = -.078 | *Z* = .409, *p* = .683 | *r* (55) = .606, *p* <.001^***^ [.408, .754] | *r_s_* (55) = .606, *p* <.001^***^ [.399, .780] |
| Adjusted AccD | .413 (.358) | .417 (.328) | | *t* (56) =-.100, *p* = .920, *d* = -.013 | *Z =* .134, *p* = .893 | *r* (55) = .577, *p* < .001^***^ [.362, .745] | *r _s_* (55) = .568, *p* < .001^***^ [.332, .735] |

*Note*. ^**^ = significant at *p* <.01, ^***^ = significant at *p* <.001. 95% confidence intervals based on 1000 bootstrap samples are reported in square brackets.

### **6e – Reaction Time Data**

For all participants with valid ECITT data in Session 2 (*N* = 121), descriptive statistics for the reaction time data (ms) for valid and correct prepotent and inhibitory trials are reported in Supplementary Table 11 below. These statistics are based on trials that were valid and correct. For a trial to be valid, the reaction time could not be < 300ms or > 5000ms, and the behavioural video coding of the trial had to be marked as valid by the offline behavioural coders (AF & KL, see SM 3a for details). Unlike analyses which we have done using this task with other age groups (e.g., Hendry et al., 2021; Holmboe et al., 2021), we did not manually correct the timing data from our 10-month-old participants and therefore, the reaction time data reported below are not in ‘clean’ form and so should be interpreted with caution.

From our experience of administering the ECITT to 10-month-old infants we know that often one of the following events may impact on infants’ reaction time:

- Infants are momentarily distracted and so reaction times can be prolonged.
- Infants touch the screen by accident (e.g., grabbing the iPad to hold it, using it for balance with one hand as they reach with the other hand, swiping their finger across the screen).
- Infants’ touch is not detected by the touch screen (e.g., if their finger is wet, or they use the side of their finger or nail to respond).

We therefore determined that it was not appropriate to use reaction time data from infants this young in our analyses, and so instead focus on accuracy data (which show solid trial type effects, as reported in the article. For further discussion of why we do not consider reaction time data from 10-month-olds valid, see Lui et al. (2021) and Hendry et al. (2021).

| Supplementary Table 11 | | | | |
| --- | --- | --- | --- | --- |
| *Reaction Time Data (ms) for Valid and Correct ECITT Trials (N = 121 participants)* | | | | |
| Trial Type | Mean | Standard Deviation | Max | Min |
| Inhibitory | 1941 | 864 | 4908 | 314 |
| Prepotent | 1831 | 907 | 4984 | 301 |
| *Note.* In line with our validity criteria, only trials with a reaction time > 300ms and < 5000ms are included here. | | | | |

# **Supplementary Materials 7 – fNIRS Data**

### **7a - Group-level analyses**

Supplementary Table 12 displays the results of a repeated measures ANOVA which investigated which channels showed significant activation in task conditions (main effect of time), as defined by a significant increase in HbO_2_ from baseline over time. Supplementary Table 13 shows the results for the HHb chromophore, where a main effect of time is indicated by a significant decrease in HHb from baseline over time. Significant results are denoted with asterisks in the tables below. Note that data from Channel 11 (right parietal cortex) and Channel 15 (left PFC) were excluded from analyses as < 70% of participants contributed data to these channels. Data from the two short separation channels (Channel 30; left PFC and Channel 34, right PFC) were also excluded.

The results of our group-level analyses are depicted in Supplementary Figures 5 and 6, which display the areas covered by the probe that showed a significant increase (HbO_2_, Figure 5) or decrease (HHb, Figure 6) from baseline in control or experimental blocks.

| Supplementary Table 12 | | | | | | |
| --- | --- | --- | --- | --- | --- | --- |
| *Significant Change in HbO_2_ Concentration (Relative to Baseline) over Time* | | | | | | |
|  |  | Left hemisphere | |  | Right hemisphere | |
| Intraparietal Sulcus | Channel | *F* | *p* | Channel | *F* | *p* |
|  | 1 | 13.045 | <.001^**^ | 7 | 25.757 | <.001^**^ |
|  | 2 | 6.205 | .001^**^ | 8 | 16.789 | <.001^**^ |
|  | 3 | 3.996 | .016^**^ | 9 | 14.259 | <.001^**^ |
|  | 4 | 6.546 | .001^**^ | 10 | 20.646 | <.001^**^ |
|  | 5 | 4.449 | .014^**^ | 12 | 4.241 | .016^**^ |
|  | 6 | 2.990 | .044^*^ |  |  |  |
| Prefrontal Cortex | 13 | 9.058 | <.001^**^ | 20 | 6.018 | .002^**^ |
|  | 14 | 21.715 | <.001^**^ | 21 | 7.765 | <.001^**^ |
|  | 16 | 9.011 | <.001^**^ | 22 | 30.804 | <.001^**^ |
|  | 17 | 30.417 | <.001^**^ | 23 | 10.211 | <.001^**^ |
|  | 18 | 9.907 | <.001^**^ | 24 | 34.045 | <.001^**^ |
|  | 19 | 9.812 | <.001^**^ | 25 | 36.921 | <.001^**^ |
|  | 27 | 3.519 | .023^**^ | 26 | 33.881 | <.001^**^ |
|  | 28 | 3.259 | .026^**^ | 31 | 11.559 | <.001^**^ |
|  | 29 | .719 | .545 | 32 | 24.829 | <.001^**^ |
|  | 35 | .905 | .414 | 33 | 13.502 | <.001^**^ |
|  | 36 | 1.509 | .225 | 37 | 7.770 | .002^**^ |
|  | 39 | .438 | .636 | 38 | 10.639 | <.001^**^ |
|  | 40 | .446 | .686 | 41 | 2.442 | .094 |
|  | 43 | .587 | .588 | 42 | 6.492 | .001^**^ |
|  | 44 | 2.094 | .129 | 45 | 3.605 | .029^**^ |
|  |  |  |  | 46 | 1.224 | .302 |

*Note.* ^*^ = significant at *p* < .05, uncorrected for multiple comparisons. To account for the violation of the sphericity assumption, the Greenhouse-Geisser corrected *p*-values are reported. ^**^ = significant at *p* <.05, corrected for 84 comparisons using the Benjamini-Hochberg procedure for controlling the false discovery rate.

| Supplementary Table 13 | | | | | | |
| --- | --- | --- | --- | --- | --- | --- |
| *Significant Change in HHb Concentration (Relative to Baseline) over Time* | | | | | | |
|  |  | Left hemisphere | |  | Right hemisphere | |
| Intraparietal Sulcus | Channel | *F* | *p* | Channel | *F* | *p* |
|  | 1 | 4.209 | .011^**^ | 7 | 7.268 | <.001^**^ |
|  | 2 | 3.044 | .038^**^ | 8 | 3.832 | .017^**^ |
|  | 3 | 8.176 | <.001^**^ | 9 | 2.861 | .056 |
|  | 4 | 5.004 | .005^**^ | 10 | 13.228 | <.001^**^ |
|  | 5 | 1.685 | .179 | 12 | .337 | .761 |
|  | 6 | 1.117 | .339 |  |  |  |
| Prefrontal Cortex | 13 | 3.819 | .014^**^ | 20 | 8.534 | <.001^**^ |
|  | 14 | 8.775 | <.001^**^ | 21 | 2.785 | .051 |
|  | 16 | 7.134 | <.001^**^ | 22 | 27.368 | <.001^**^ |
|  | 17 | 16.503 | <.001^**^ | 23 | 7.300 | <.001^**^ |
|  | 18 | 10.422 | <.001^**^ | 24 | 20.272 | <.001^**^ |
|  | 19 | 12.864 | <.001^**^ | 25 | 20.585 | <.001^**^ |
|  | 27 | 8.479 | <.001^**^ | 26 | 31.379 | <.001^**^ |
|  | 28 | 10.361 | <.001^**^ | 31 | 4.465 | .006^**^ |
|  | 29 | 5.547 | .001^**^ | 32 | 24.005 | <.001^**^ |
|  | 35 | 2.879 | .037^**^ | 33 | 19.668 | <.001^**^ |
|  | 36 | 4.265 | .007^**^ | 37 | .908 | .422 |
|  | 39 | 12.444 | <.001^**^ | 38 | 4.787 | .004^**^ |
|  | 40 | 12.389 | <.001^**^ | 41 | 5.442 | .002^**^ |
|  | 43 | 5.792 | .003^**^ | 42 | 5.225 | .004^**^ |
|  | 44 | 2.040 | .132 | 45 | 8.818 | <.001^**^ |
|  |  |  |  | 46 | 6.374 | .003^**^ |

*Note.* ^**^ = significant at *p* <.05, corrected for 84 comparisons using the Benjamini-Hochberg procedure for controlling the false discovery rate. To account for the violation of the sphericity assumption, the Greenhouse-Geisser corrected *p*-values are reported.

| Supplementary Figure 5 |
| --- |
| *Group-level T-statistic Images of Changes in HbO_2_ Concentration Relative to Baseline* |
| 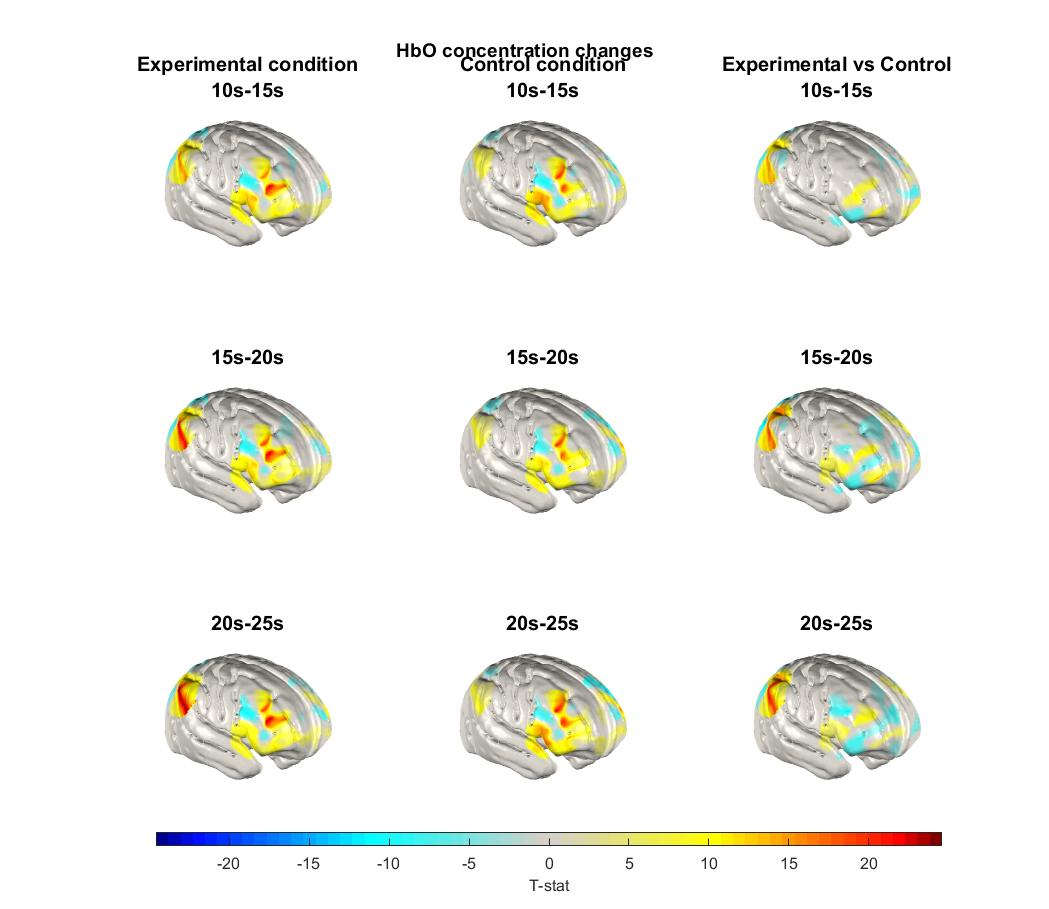 |
| *Note.* Group-level T-statistic images of changes in oxygenated haemoglobin concentration changes in response to the experimental (left column) and control (middle column) blocks across 10 - 25 seconds of the block time course relative to a 2 second pre-stimulus baseline. Using this approach, all displayed T-statistic values are significantly different across block type (two sample *t*-test) at the alpha level of *p* < .01. Right column: Group-level T-statistic images of the contrast in HbO_2_ concentration changes between the experimental and control blocks (experimental – control). All displayed T-statistic values are significantly different across block types (paired *t*-test) at the alpha level of *p* < .01. Images are displayed in the space of a cortical surface derived from averaged structural MRI data of a 12-month-old cohort of infants (Shi et al. (2011)). |

| Supplementary Figure 6 |
| --- |
| *Group-level T-statistic images of changes in HHb Concentration Relative to Baseline* |
| 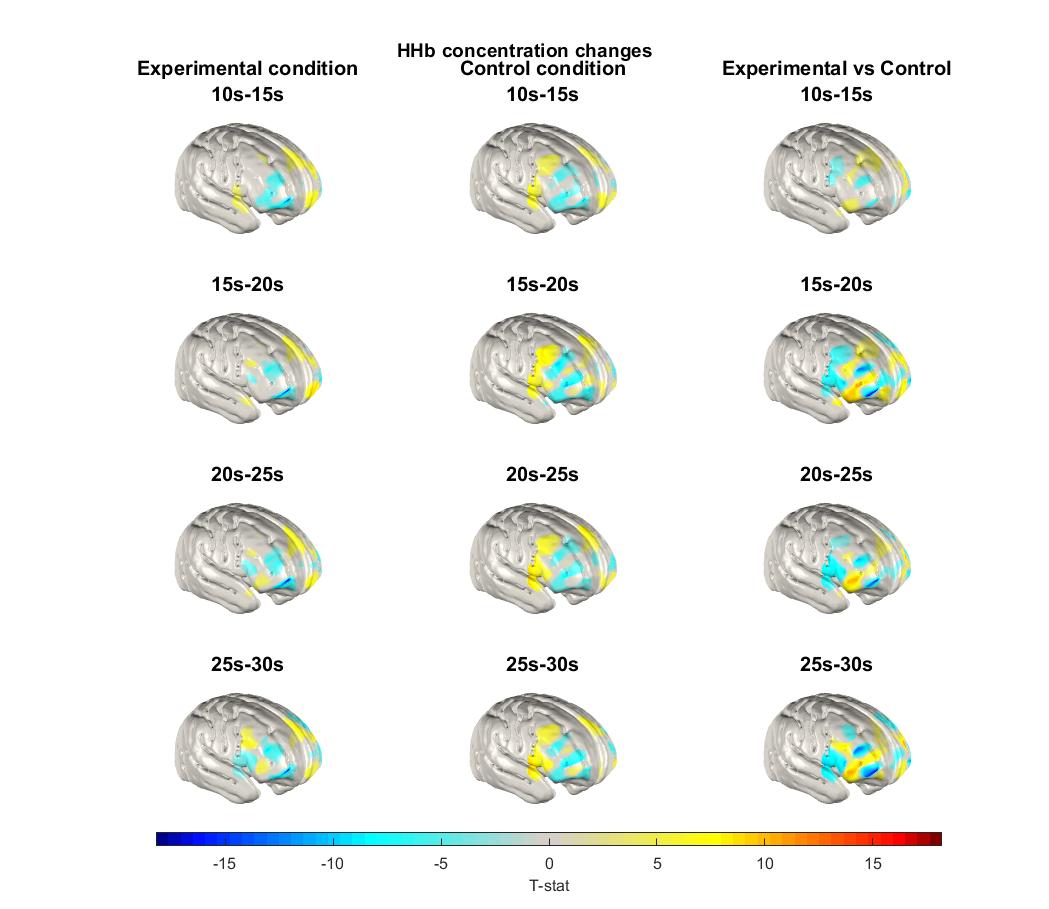 |
| *Note.* Group-level T-statistic images of changes in deoxygenated haemoglobin (HHb) concentration changes in response to the experimental (left column) and control (middle column) blocks across 10-30 seconds of the block time course relative to a 2 second pre-stimulus baseline. Using this approach, all displayed T-statistic values are significantly different across block type (independent *t*-test) at the alpha level of *p* < .01. Right column: Group-level T-statistic images of the contrast in HHb concentration changes between the experimental and control blocks(experimental – control). All displayed T-statistic values are significantly different across block types (two sample *t*-test) at the alpha level of *p* < .01. Images are displayed in the space of a cortical surface derived from averaged structural MRI data of a 12-month-old cohort of infants (Shi et al. (2011). Since we found no significant block-type HHb effects in parietal regions, this figure only shows activation in frontal regions. |

### **7b - Justification for the exclusion of Channel 32 from the right PFC cluster (HbO_2_)**

Following our group-level analyses, we identified Channel 32 (right PFC) as showing a significant time × block type interaction for HbO_2_ (see Table 1 of article). We decided not to include the HbO_2_ data from Channel 32 in the right dorsolateral PFC pair (Channels 25 and 26) for the investigation of the time course of the block type effect. This is because, following inspections of the haemodynamic response function (HRF) plots of the three frontal channels, we found that whilst the HbO_2_ signal for Channels 25 and 26 looked visually very similar the HbO_2_ signal for Channel 32 did not appear to follow the same pattern (see Figure 5 in article). Further, the location of Channel 32, whilst proximal to Channels 25 and 26 (see Figure 5 in main article and Supplementary Figure 1), appears to be in a more anterior part of the right PFC (specifically, the orbitofrontal cortex) rather than in the dorsolateral regions of the right PFC in which Channels 25 and 26 are positioned (specifically, the right middle frontal gyrus). Since orbital regions of the right PFC have not consistently been associated with response inhibition within the literature, we therefore decided to examine the time course of the block type effect in Channel 32 separately from the other two frontal channels, see also the ‘Exploratory analysis’ section of our pre-registration where this decision is discussed: <https://osf.io/qs4h8>.

### **7c - Individual Differences Analyses**

This section refers to the individual differences analyses that we pre-registered (<https://osf.io/qs4h8>) following our group level analysis of the fNIRS data (reported in Section 3.2 of the article). Since we were unaware of the distribution of the neural indices, we had pre-registered that we would conduct both parametric (Pearson’s) and non-parametric (Spearman’s) correlational analyses and expected convergence in the results. Following pre-registration, all variables were tested for the assumptions of parametric tests and the results are reported below.

According to the Shapiro-Wilk test of normality, data from the HbO_2_ right lateral PFC variable (bins 3 – 5) and the HHb data from Channel 25 were normally distributed, respectively; *p* = .100 and *p* = .305, however the inhibitory score variable and the HbO_2_ data from the right parietal cluster (bins 3 – 5) were not normally distributed; respectively, *p* = .029 and *p* = .011. The inhibitory score variable was negatively skewed but within an acceptable level (*z* = .347) and the HbO_2_ right parietal cluster (bins 3 – 5 variable) was positively skewed within an acceptable level (*z* = 3.01), according to the parameters outlined by Kim (2013). As such, most variables were skewed within acceptable limits, however, in accordance with the pre-registration, we report the non-parametric correlations below.

Results of the Spearman’s rho correlational analyses revealed that there was no significant association between individual differences in infants’ inhibitory score and HbO_2_ activation in the right lateral PFC pair; *r*_s_ (57) = -.070, *p* = .298, [*CI* = -.313, .158], or in the right parietal pair; *r_s_* (53) = .071, *p* = .304, [*CI* = .232, .336]. No significant association was found between HHb concentration difference from 10 – 25 seconds in Channel 25 (right PFC) and inhibitory performance using Spearman’s rho correlation; *r*_s_ (54) = .216, *p* = .055, [*CI* = -.050, .444]. This contrasts with the results of the Pearson’s correlational analysis which did indicate a significant nominal association (*p* = .029), reported in Section 3.3. of the article.

### **7d - Exploratory Analysis**

In accordance with our pre-registered exploratory analyses, (<https://osf.io/qs4h8>) we performed (two-tailed) correlational analyses in order to examine whether the difference in HHb concentration difference across time bins 4 – 6 (15 – 30 seconds of the block time course) in a pair of channels in the right anterior PFC (Channels 32 and 33) was associated with infants’ response inhibition performance. The results are described in Section 3.3.1 of the article but see Supplementary Figure 7 for the scatterplot of this association.

According to the Shapiro-Wilk test, the HHb concentration difference variable was not normally distributed (*p* = .018), but the z-score of the skew indicates an acceptable level of positive skew (*z* = 2.55; Kim, 2013). The results from the two correlation methods did not converge. Results of the Pearson’s correlation indicated that there was no significant association between HHb concentration difference in the orbital PFC and inhibitory performance (reported in Section 3.3.1 of article, and see Supplementary Figure 7), whereas Spearman’s correlation indicated that a significant weak negative association was present; *r_s_* (57) = -.281, *p* = .031, [*CI* = -.506, -.011]. As in the main article, we conducted further exploratory analyses (two-tailed) at the channel-level across the same time course. We found no evidence of a significant association between HHb concentration difference in Channel 32 and inhibitory performance using Pearson’s (*p* = .537) or Spearman’s correlations (*p* = .527) but did find a significant association between HHb concentration difference in Channel 33 and inhibitory performance using both Pearson’s (reported in Section 3.3.1 of article) and Spearman’s correlations; *r_s_* (57) = -.295, *p* = .023, [*CI* = -.519, -.054]. Supplementary Figure 8 illustrates this significant negative association.

| **Supplementary Figure 7** |
| --- |
| *Association between the HHb difference score in right anterior PFC, Bins 4 – 6 and Inhibitory Score* |
| 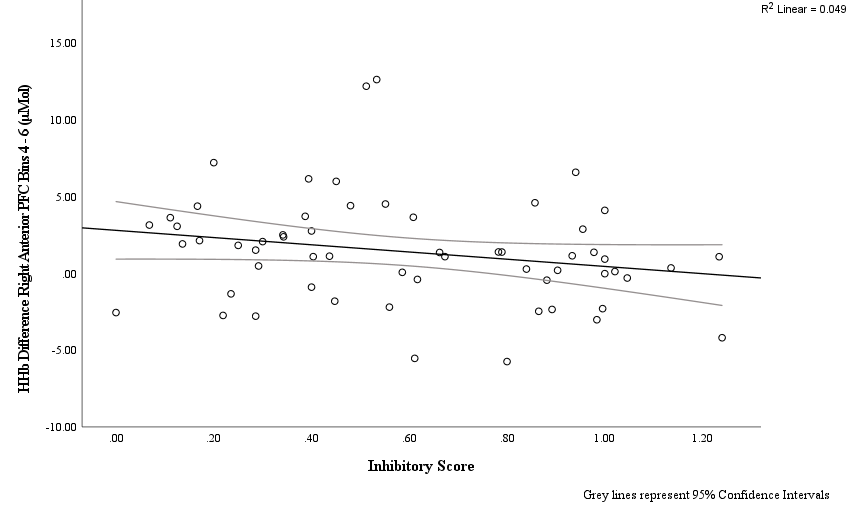 |
| *Note.* A larger inhibitory score is indicative of better response inhibition performance, and a larger HHb difference score indicates that there is a larger decrease in HHb in experimental compared to control conditions. The HHb concentration difference was calculated (per time bin) as: average baseline-corrected HHb concentration in control blocks minus average baseline-corrected HHb concentration in experimental blocks. The HHb difference for Channels 32 and 33 were averaged for each time bin (4 – 6; 15 – 30 seconds of the block time course). The average HHb for each time bin was then averaged to create the measure ‘HHb difference right anterior PFC, bins 4 – 6’. |

| **Supplementary Figure 8** |
| --- |
| *Association between the HHb difference score in Channel 33 (Bins 4 – 6) and Inhibitory Score* |
| 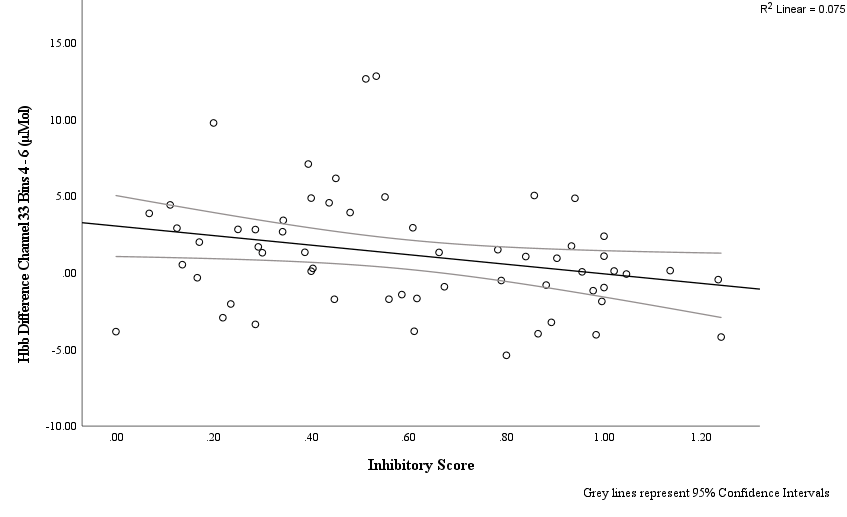 |
| *Note.* A larger inhibitory score is indicative of better response inhibition performance, and a larger HHb difference score indicates that there is a larger decrease in HHb in experimental compared to control conditions. The HHb concentration difference was calculated as: average baseline-corrected HHb concentration in control blocks minus average baseline-corrected HHb concentration in experimental blocks. This was averaged across time bins 4 – 6 (15 – 30 seconds of the block time course) for Channel 33. |

### **7e – Additional Group-Level Analysis (Including Time-Bins 8 and 9)**

As detailed in the main article (Section 2.5.2), we decided to exclude time-bins 8 and 9 (35 - 45s of the block time course) from our analyses of the fNIRS data. When investigating the duration of each individual block completed by each of the participants with valid fNIRS data (N = 59), we found that the minimum average duration of control blocks was 31 seconds. This means that all participants contributed control block data of at least 31 seconds (time-bin 7; 30 – 35 seconds), however only 49 participants had a mean control block duration of between 35 – 40 seconds (time-bin 8), reducing to 26 participants who had a mean control block duration that exceeded 40 seconds (time-bin 9). For experimental blocks, the minimum average duration was 36 seconds (time-bin 8; 35 – 40 seconds), meaning that all participants contributed experimental block data up until time-bin 8. However, only 43 participants had a mean experimental block duration that exceeded 40 seconds (time-bin 9). As such, due to the variable block duration (which is contingent on the duration of infants’ responses), these last time-bins are capturing baseline data from some participants and task data from others, rendering these time-bins unsuitable for analysis. For transparency, we provide a report (below) of the fNIRS results when time-bins 8 and 9 are included in analyses. The analyses conducted were the same as reported in the main article.

Repeated measures ANOVAs identified that 41 (of 42 channels included in the analysis) demonstrated a main effect of time; a significant increase in HbO_2_ and/or decrease in HHb from baseline over time. A total of 31 channels showed a significant HbO_2_ increase (from baseline) over time, of which 27 also showed a significant HHb decrease from baseline over time, and 4 showed only a significant HbO_2_ increase. A total of 10 channels showed only a significant HHb decrease and as such, a total of 37 channels showed a significant main effect of time in HHb. The Benjamini-Hochberg procedure for controlling the false discovery rate (FDR; Benjamini & Hochberg, 1995) was conducted for 84 comparisons. All channels retained significance (except Channel 6, HbO_2_ only, which was then excluded from further analyses). Discrepancies between these results and the results reported in the main article are as follows: Channels 9, 21 and 44 now show a significant main effect of time in HHb, where previously it was not significant. Channel 44 now shows a significant main effect of time in HbO_2_, and Channel 45 no longer shows a significant main effect of time in HbO_2._

From repeated measures analyses with these 41 channels, we identified four channels in the right PFC (Channels 24, 25, 26 and 32) and two in the right parietal cortex (Channels 10 and 12) that showed a significantly greater baseline-corrected HbO_2_ increase in experimental compared to control blocks (main effect of block type), and four channels in the right PFC (Channels 25, 32, 33 and 46) that showed a significantly greater baseline-corrected decrease in HHb in experimental compared to control blocks. None of these effects survived correction for the FDR.

Discrepancies between these results and the results reported in the main article are as follows: A significant time × block type interaction was found in HbO_2_ for Channel 24 (*p* = .042), where previously this was non-significant (*p* = .098). Paired *t*-tests (control vs. experimental, HbO_2_) for each time bin in Channel 24 revealed that there was a significantly (*p* = .010) larger HbO_2_ increase in experimental compared to control blocks from 40 – 45 seconds (time bin 9). As previously discussed, it is not meaningful to interpret this effect since this time period is contaminated with signal from both task and baseline blocks. Similarly, a significant time × block type interaction was found in HHb for Channel 46 (*p* = .023), where previously no significant effect was found (*p* = .163). Paired *t­*-tests revealed a significant difference in HHb concentration between block types in time bins 1 (0 – 5 seconds) and 9 (40 – 45 seconds), however this effect was in the opposite direction than expected as HHb concentrations were greater in experimental compared to control blocks and so again, it is not meaningful to interpret this effect.

When investigating the time course of the significant effects at the channel level (in the same channels that we had previously identified in our analysis reported in the main article), we found a significant difference in HbO_2_ concentration between block types in time-bins 8 and 9 (35 – 45 seconds) in all four channels (lowest *p* = .001, highest *p* = .033). Similarly, we found a significant HHb concentration difference between block types in time-bin 8 (Channel 32, *p* = .048), and 9 (Channel 25, *p* = .035). As discussed, it is not sensible to interpret the effects in time-bins 8 and 9 as it is not possible to disentangle the signal obtained from task blocks and that obtained during the baseline periods. Therefore, by trimming our analysis time window to 35 seconds (time bins 1 – 7), we are interpreting data only collected during task blocks and as such, meaningful conclusions can be drawn with greater confidence.

# **References**

Benjamini, Y., & Hochberg, Y. (1995). Controlling the false discovery rate : a practical and powerful approach to multiple testing. *Journal of the Royal Statistical Society*, *57*(1), 289–300. https://doi.org/10.1111/j.2517-6161.1995.tb02031.x

Brigadoi, S., & Cooper, R. J. (2015). How short is short? Optimum source–detector distance for short-separation channels in functional near-infrared spectroscopy. *Neurophotonics*, *2*(2), 025005. <https://doi.org/10.1117/1.NPh.2.2.025005>

Brigadoi, S., Galderisi, A., Pieropan, E., Cooper, R. J., Cutini, S., Baraldi, E., Cobelli, C., Dell’Acqua, R., Sparacino, G., & Trevisanuto, D. (2019). Mapping hemodynamic changes during hypoglycemia in the very preterm neonatal brain: Preliminary results. *Optics InfoBase Conference Papers*, *Part F142*-*ECBO 2019*, 11074_13. <https://doi.org/10.1117/12.2526974>

Collins-Jones, L. H., Arichi, T., Poppe, T., Billing, A., Xiao, J., Fabrizi, L., Brigadoi, S., Hebden, J. C., Elwell, C. E., & Cooper, R. J. (2021). Construction and validation of a database of head models for functional imaging of the neonatal brain. *Human Brain Mapping*, *42*(3), 567–586. <https://doi.org/10.1002/hbm.25242>

Emberson, L. L., Crosswhite, S. L., Goodwin, J. R., Berger, A. J., & Aslin, R. N. (2016). Isolating the effects of surface vasculature in infant neuroimaging using short-distance optical channels: a combination of local and global effects. *Neurophotonics, 3(*3), 031406. DOI: <https://doi.org/10.1117/1.NPh.3.3.031406>

Fang, Q., & Boas, D. A. (2009). Tetrahedral mesh generation from volumetric binary and grayscale images. *Proceedings - 2009 IEEE International Symposium on Biomedical Imaging: From Nano to Macro, ISBI 2009*, 1142–1145. <https://doi.org/10.1109/ISBI.2009.5193259>

Frijia, E. M., Billing, A., Lloyd-Fox, S., Rosas, E. V., Collins-Jones, L., Crespo-Llado, M. M., ... & Cooper, R. J. (2021). Functional imaging of the developing brain with wearable high-density diffuse optical tomography: a new benchmark for infant neuroimaging outside the scanner environment. NeuroImage, 225, 117490. <https://doi.org/10.1016/j.neuroimage.2020.117490>

Holmboe K, Larkman C, de Klerk C, Simpson A, Bell MA, Patton L, et al. (2021) The early childhood inhibitory touchscreen task: A new measure of response inhibition in toddlerhood and across the lifespan. *PLoS ONE,* *16*(12): e0260695. <https://doi.org/10.1371/journal.pone.0260695>

Huppert, T. J., Diamond, S. G., Franceschini, M. A., & Boas, D. A. (2009). HomER: A review of time-series analysis methods for near-infrared spectroscopy of the brain. *Applied Optics*, *48*(10). https://doi.org/10.1364/AO.48.00D280

Jenkinson, M., Pechaud, M., & Smith, S. (2005). BET2-MR-Based Estimation of Brain, Skull and Scalp Surfaces. *Human Brain Mapping*, *17*(2), 143–155. www.fmrib.ox.ac.uk/analysis/research/bet

Kim, H. Y. (2013). Statistical notes for clinical researchers: assessing normal distribution (2) using skewness and kurtosis. *Restorative dentistry & endodontics*, *38*(1), 52-54. https://doi.org/10.5395/rde.2013.38.1.52

Shi, F., Yap, P.-T., Wu, G., Jia, H., Gilmore, J. H., Lin, W., & Shen, D. (2011). Infant Brain Atlases from Neonates to 1- and 2-Year-Olds. *PLoS ONE*, *6*(4), e18746. https://doi.org/10.1371/journal.pone.0018746

Zhou, X., Sobczak, G., McKay, C. M., & Litovsky, R. Y. (2020). Comparing fNIRS signal qualities between approaches with and without short channels. *PloS One*, *15*(12), e0244186. https://doi.org/10.1371/journal.pone.0244186
